# The unprecedented diversity of UGT94-family UDP-glycosyltransferases in *Panax* plants and their contribution to ginsenoside biosynthesis

**DOI:** 10.1038/s41598-020-72278-y

**Published:** 2020-09-21

**Authors:** Chengshuai Yang, Chaojing Li, Wei Wei, Yongjun Wei, Qunfang Liu, Guoping Zhao, Jianmin Yue, Xing Yan, Pingping Wang, Zhihua Zhou

**Affiliations:** 1 CAS-Key Laboratory of Synthetic Biology, CAS Center for Excellence in Molecular Plant Sciences/Shanghai Institute of Plant Physiology and Ecology, Chinese Academy of Sciences, Shanghai, 200032 China; 2 Institute of Synthetic Biology, Shenzhen Institutes of Advanced Technology, Chinese Academy of Sciences, Shenzhen, 518055 China; 3 State Key Laboratory of Drug Research, Shanghai Institute of Materia Medica, Chinese Academy of Sciences, Shanghai, 201203 China; 4 University of Chinese Academy of Sciences, Beijing, 100049 China

**Keywords:** Biochemistry, Biotechnology, Molecular biology, Plant sciences

## Abstract

More than 150 ginsenosides have been isolated and identified from *Panax* plants. Ginsenosides with different glycosylation degrees have demonstrated different chemical properties and bioactivity. In this study, we systematically cloned and characterized 46 UGT94 family UDP-glycosyltransferases (UGT94s) from a mixed *Panax ginseng*/callus cDNA sample with high amino acid identity. These UGT94s were found to catalyze sugar chain elongation at C3-O-Glc and/or C20-O-Glc of protopanaxadiol (PPD)-type, C20-O-Glc or C6-O-Glc of protopanaxatriol (PPT)-type or both C3-O-Glc of PPD-type and C6-O-Glc of PPT-type or C20-O-Glc of PPD-type and PPT-type ginsenosides with different efficiencies. We also cloned 26 and 51 UGT94s from individual *P*. *ginseng* and *P*. *notoginseng* plants, respectively; our characterization results suggest that there is a group of UGT94s with high amino acid identity but diverse functions or catalyzing activities even within individual plants. These UGT94s were classified into three clades of the phylogenetic tree and consistent with their catalytic function. Based on these UGT94s, we elucidated the biosynthetic pathway of a group of ginsenosides. Our present results reveal a series of UGTs involved in second sugar chain elongation of saponins in *Panax* plants, and provide a scientific basis for understanding the diverse evolution mechanisms of UGT94s among plants.

## Introduction

Plants collectively synthesize more than 200,000 natural products, a significant proportion of which are glycosylated^1^. Glycosylation alters the hydrophilicity, stability, and subcellular localization of natural products, and thus, their chemical properties and bioactivities^2^. Glycosylation of natural products in plants is catalyzed by glycosyltransferases. In the glycosylation of natural products, the sugar donors are usually uridine diphosphate (UDP)-activated monosaccharides; therefore, these glycosyltransferases are termed as UDP-glycosyltransferases (UGTs)^3^.

Ginsenosides, mainly comprising protopanaxadiol (PPD)- and protopanaxatriol (PPT)-type saponins, are the main bioactive constituents of *Panax* plants, including *P*. *ginseng* and *P*. *notoginseng*^4–7^. More than 150 structurally diverse ginsenosides have been isolated from *Panax* species; they are conjoined with one to six sugar units, and up to three at the C3 and C20 sites of PPD-type ginsenosides or the C6 and C20 sites of PPT-type ginsenosides^8^. The number, positions, and types of sugar units, mainly comprising glucose, xylose, arabinose, and rhamnose, contribute greatly to ginsenoside diversity^8^. Among most reported ginsenosides, glucose preferentially links with the dammarane skeleton, with all four sugar unit types appearing in the second unit of the sugar chain attached to the dammarane skeleton. The diversity and combination of sugar units allow ginsenosides to exhibit diverse bioactivities. Therefore, it is also of high importance for ginsenosides drug research to elucidate the mechanisms of sugar chain elongation of ginsenosides.

In previous studies, we characterized several UGTs from *P. ginseng.* PgUGT71A53 (UGTPg1), PgUGT74AE4 (UGTPg45), and PgUGT71A54 (UGTPg100) or PgUGT71A55 (UGTPg101) were found to control glycosylation of the first sugar unit toward the C20-OH, C3-OH, and C6-OH sites of PPD or PPT, respectively^9–11^. The biosynthetic pathways of five ginsenosides, PPD-type ginsenosides CK and Rh2 and PPT-type ginsenosides Rh1, F1, and Rg1 have been elucidated. However, most ginsenosides among *Panax* plants typically harbor two or more sugar units at a single position of the PPD or PPT skeleton, suggesting that more UGTs are responsible for sugar chain elongation of ginsenosides; these UGTs remain uncharacterized. Although a purified UGT from *P. notoginseng* has been characterized to catalyze Rd to produce Rb1 by sugar chain elongation, its encoding gene was unknown at that time^12^. We previously characterized PgUGT94Q2 (UGTPg29) as the first sequenced *Panax* UGT to exhibit sugar chain elongation activity (sugar–sugar UGT). PgUGT94Q2 has subsequently been demonstrated to belong to the UGT94 family and to transfer a glucose moiety to the C3-O-Glc site of Rh2 to produce Rg3^10,13^. However, the sugar–sugar UGTs responsible for transferring a glucose unit to the C6-O-Glc of PPT-type ginsenosides and C20-O-Glc of PPD- and PPT-type ginsenosides, as well as transferring a xylose, arabinose, or rhamnose unit to the C3, C6, or C20 glucose moiety of *Panax* plants, have not yet been reported.

Large quantities of low-molecular-weight natural products have been linked with two to four sugar units at a single position in plants. Accordingly, sugar–sugar UGTs, which are responsible for transferring a sugar group to an existing sugar moiety in natural products, may be diverse^14^. However, only a limited number of sugar–sugar UGTs have been characterized, mainly from the UGT94, UGT73, UGT79, and UGT91 families^14–23^. These sugar–sugar UGTs could add a glucosyl/glucuronosyl/xylose/rhamnosyl/galactosyl molecule to an existing sugar moiety in natural products, including flavonoids and terpenoids. It has been reported that some UGTs from the UGT91 family (UGT91H4 and UGT91H9) can transfer a rhamnosyl or glucosyl moiety to the third position of the sugar chain^22,23^. In the past few decades, research has been focused on cloning and characterizing UGTs that glycosylate the OH and/or COOH group of natural products. Therefore, more effort should be devoted to mining and characterizing sugar–sugar UGTs^14^.

In this study, we systematically cloned and characterized a series of UGT94s with high amino acid identity from *P*. *ginseng* and *P*. *notoginseng*; these UGT94s are sugar–sugar UGTs being responsible for second sugar chain elongation in ginsenosides. Our results elucidated possible biosynthetic pathways of a group of ginsenosides in *Panax* plants, and contributed to the elucidation of a ginsenoside biosynthetic pathway network, as well as the discovery of sugar–sugar UGTs involved in saponin biosynthesis in other plants. The cloning and characterization of these UGT94s provides a basis for setting up a platform to synthesize a series of ginsenosides from glucose using microbial cell factories.

## Results and discussion

### Cloning and functional characterization of *P. ginseng* UGT genes with high amino acid identity to PgUGT94Q2

In one of our previous studies, the UDP-glycosyltransferase PgUGT94Q2 was cloned and characterized to selectively transfer a glucose moiety to the C3-O-Glc of Rh2 to form Rg3^10^. Some of the UGT fragments (ORFs) in our established *Panax* cDNA database were found to be aligned to *PgUGT94Q2* with high amino acid identity (> 75%)^10^. Four pairs of full-length primers were designed based on these fragments and applied to clone UGT genes using a mixed cDNA sample of a *P*. *ginseng* plant and callus as the template. In total, 303 clones were picked up and sequenced (Table S1). The deduced amino acids of the 303 UGTs were all predicted to belong to the UGT94 family^24^, and were assigned into 46 individual UGT units (Table S1). The deduced amino acids of the 46 *PgUGT94s* shared high amino acid identity, ranging from 85.87% to 99.78% (Table S2).

Of the 46 *PgUGT94s*, 11 could be cloned by two or more pairs of primers, among which *PgUGT94Q2* and *PgUGT94Q5-V1* were cloned by the four pairs of primers. Multiple clones were detected for 19 *PgUGT94s*, whereas only one clone was detected for the remaining 27. In total, the deduced amino acid sequences of 84 and 49 clones were identical to those of *PgUGT94Q2* and *PgUGT94Q5-V1*, respectively (Table S1), demonstrating that they could be the major UGT94 units in *P*. *ginseng*.

**Figure 1:**
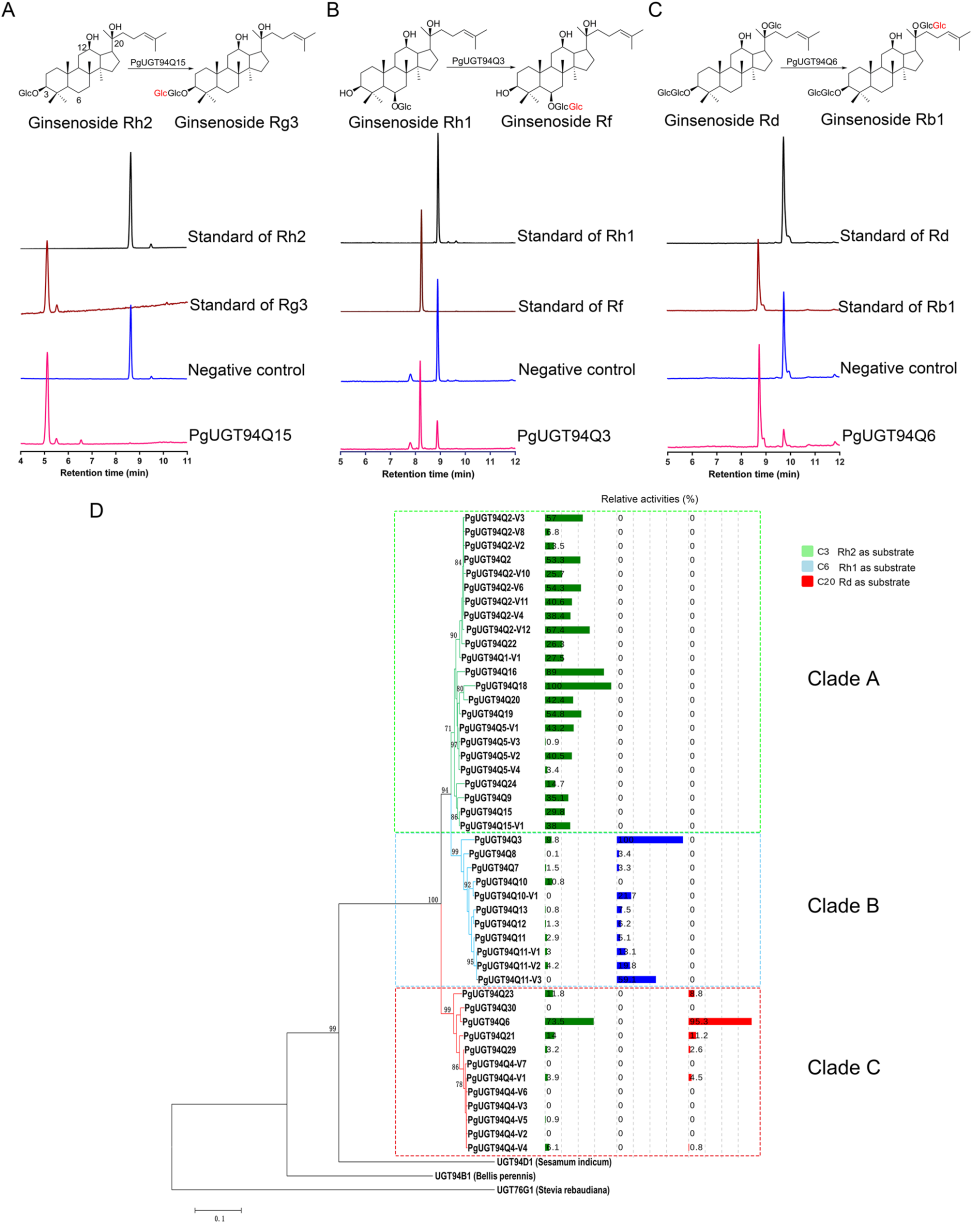
Cloning and functional characterization of *Panax ginseng* UGT94s with high identity to PgUGT94Q2. (**A**) High-performance liquid chromatography (HPLC) analysis of in vitro reactions of PgUGT94Q15 toward Rh2 with UDP-glucose as the sugar donor. (**B**) HPLC analysis of in vitro reactions of PgUGT94Q3 toward Rh1 with UDP-glucose as the sugar donor. (**C**) HPLC analysis of in vitro reactions of PgUGT94Q6 toward Rd with UDP-glucose as the sugar donor. (**D**) Phylogenetic analyses of deduced amino acid sequences of *PgUGT94*s from mixed cDNA of a *P*. *ginseng* plant and callus with other characterized plant UGTs, and relative activities of functional PgUGT94s. The relative enzyme activities of PgUGT94Q18, PgUGT94Q3 and PnUGT94Q25-V2 (with the highest enzyme activity toward substrate Rh2, Rh1 and Rd, respectively) were designated as 100%. The relative enzyme activities of the left UGT94s toward substrate Rh2, Rh1 and Rd were standardized by comparing with the three representative ones, respectively. Only bootstrap values above 70% are shown in the tree.

For the in vitro enzymatic functional assay, the PPD-type saponins CK, Rh2, and Rd, as well as the PPT-type saponins Rh1 and F1, were selected as putative sugar acceptors (substrates). Catalytic function assays of the 46 PgUGT94s were performed using crude cell extracts of the individual expressed PgUGT94s with UDP-glucose as the putative sugar donor. The results showed that 41 of the 46 PgUGT94s catalyzed the tested substrates, of which 39 PgUGT94s catalyzed Rh2 to produce Rg3 (Fig. 1A and D; Figs. S1A, S7A, S7B, and S8A; Table S1). This result indicates that more than four-fifths of the PgUGT94s exhibit sugar chain elongation activity toward C3-O-Glc of PPD-type saponins, with different efficiencies. We also found that 10 PgUGT94s had catalytic activity toward Rh1 to form Rf (Fig. 1B and D; Figs. S1B, S7C, S7D, S8B, and S8C; Table S1), demonstrating that they can extend the sugar chain at C6-O-Glc of PPT-type saponins. Five PgUGT94s converted CK to gypenoside LXXV (Figs. S1D, S2A, S7I, and S8F-I), Rd to Rb1 (Fig. 1C and D; Figs. S1C, S7E, S7F, and S8D; Table S1), and F1 to notoginsenoside U (Figs. S1H, S2D, S7M, and S8E), suggesting that these five PgUGT94s are able to glycosylate the C20-O-Glc of both PPD- and PPT-type saponins (Table S1). Eight PgUGT94s demonstrated sugar chain elongation for both C3-O-Glc of PPD-type ginsenosides and C6-O-Glc of PPT-type saponins, and five PgUGT94s exhibited sugar chain elongation for both C3-O-Glc and C20-O-Glc of PPD-type ginsenosides (Fig. 1D; Table S1). In addition, PgUGT94Q15 and PgUGT94Q15-V1, the sugar–sugar UGTs for C3-O-Glc of PPD-type ginsenosides, showed sugar chain elongation at C3-O-Glc of 3-O-*β*-Glc oleanolic acid (3GOA) to form 3-*O*-[*β*-D-glucopyranosyl-(1 → 2)-*β*-D-glucopyranosyl]-oleanolic acid (Fig. S2C, S7L and S8J-O), which has yet to be isolated and detected from *Panax* plants. PgUGT94Q15-V1 catalyzed calunduloside E to zingibroside R1 by transferring a glucose moiety at C3-O-GlcA (Figs. S1I, S2E, S7J, and S7K). Among the remaining five PgUGT94s, no activity was detected in any of the used substrates (Fig. 1D; Fig. S1; Table S1).

The 46 PgUGT94s were classified into three clades of the phylogenetic tree, consistent with their functions and activities (Fig. 1D). All of the 23 PgUGT94s in clade A exhibited sugar chain elongation activity only at C3-O-Glc of PPD-type saponins, among which PgUGT94Q18 had the highest activities to Rh2, the relative activities of others 22 PgUGT94s ranged from 0.9% to 89% of PgUGT94Q18. Most of the 11 PgUGT94s in clade B exhibited sugar chain elongation activity at both C3-O-Glc of PPD-type and C6-O-Glc of PPT-type saponins, among which PgUGT94Q3 had the highest activities at C6-O-Glc of Rh1 and C3-O-Glc of Rh2. The remaining PgUGT94s in clade B exhibited lower activities to the tested substrates; PgUGT94Q10 had no activity, but PgUGT94Q10-V1 and PgUGT94Q11-V3 had low activity to PPT-type substrates. The five PgUGT94s without activity to the tested substrates and seven functional PgUGT94s clustered in clade C. All of the seven functional PgUGT94s in clade C demonstrated elongation activity at C3-O-Glc of PPD-type saponins, of which five also showed elongation activity at C20-O-Glc of PPD- and PPT-type saponins. Among them, PgUGT94Q6 had the highest activities both towards C3-O-Glc of Rh2 and C20-O-Glc of Rd; the remaining functional PgUGT94s only showed very weak substrate activities.

Several UGTs of the UTG94 family have previously been reported to catalyze sugar chain elongation of natural products including monoterpene, triterpenoid mogrosides, flavonoids, and lignans^14,16,18–20^. For example, CaUGT3 from *Catharanthus roseus* was found to control sugar chain elongation from the second to fourth positions of the sugar chain^14^. UGT94P1 from *Camellia sinensis*, UGT94B1 from *Bellis perennis*, and PgUGT94Q2 from *P*. *ginseng* have been reported as sugar–sugar UGTs, transferring the second sugar moiety to the sugar chain^10,19,20^. These findings support speculation that the UGT94 family participates extensively in sugar chain elongation in natural products.

### UGT94 diversity in a single *P. ginseng* plant

We cloned diverse PgUGT94s with high amino acid identity from the cDNA of a mixed *P*. *ginseng* sample (85.87%–99.78%). To explore whether similar diversity exists at the level of a single *P*. *ginseng* plant, we cloned *UGT94*s from the cDNA of the leaves and roots of an individual *P*. *ginseng* plant using the same four pairs of primers. A total of 228 and 184 clones were obtained from *P*. *ginseng* leaf and root cDNA, respectively (Table S3). Among the 228 clones derived from leaf cDNA, 217 were identical to four detected *PgUGT94*s from the *P*. *ginseng* mixed cDNA (i.e., *PgUGT94Q2*, *Q3*, *Q4-V1*, and *Q5-V3*). The remaining 11 clones resulted in nine new *PgUGT94*s. Among the 184 clones derived from root cDNA, 175 were identical to nine detected *PgUGT94s* from the mixed cDNA (i.e., *PgUGT94 Q1-V1*, *Q2*, *Q3*, *Q4-V1*, *Q4-V2*, *Q5-V3*, *Q22*, *Q23*, and *Q29*). The remaining nine clones resulted in eight new UGT94s that have been not previously obtained (Table S3).

**Figure 2:**
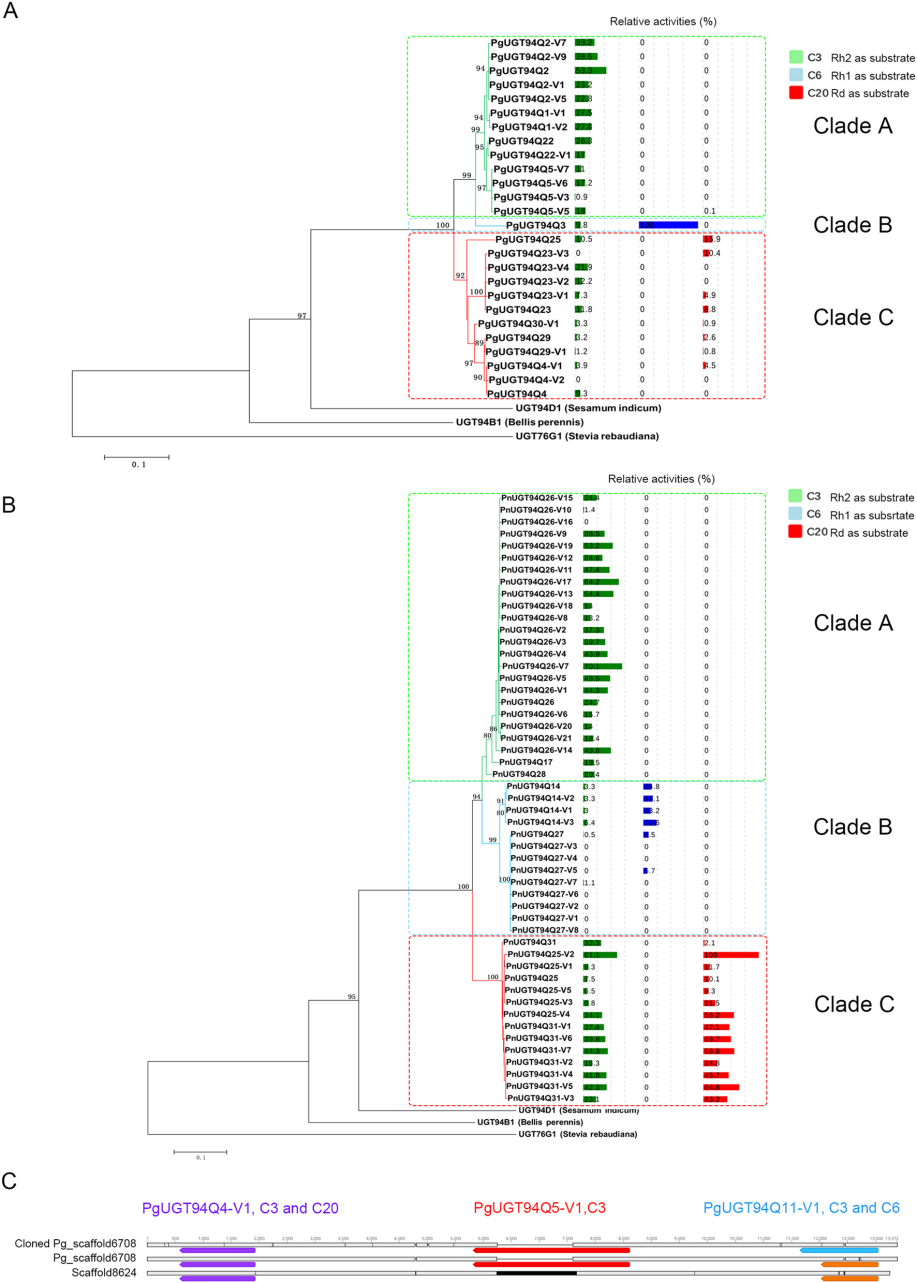
Diversity analyses of UGT94s from individual *Panax* plants. (**A**) Phylogenetic analyses of deduced amino acid sequences of *PgUGT94*s from an individual *P*. *ginseng* plant with other characterized plant UGTs and relative activity of functional UGT94s. The relative enzyme activities of PgUGT94Q18, PgUGT94Q3 and PnUGT94Q25-V2 (with the highest enzyme activity toward substrates Rh2, Rh1 and Rd, respectively) were designated as 100%. The relative enzyme activities of the left UGT94s toward substrates Rh2, Rh1 and Rd were standardized by comparing with the three representative ones, respectively. Only bootstrap values above 70% are shown in the tree. (**B**) Phylogenetic analyses of deduced amino acid sequences of *PnUGT94*s from an individual *P*. *notoginseng* plant with other characterized plant UGTs and relative activity of functional UGT94s. The relative enzyme activities of PgUGT94Q18, PgUGT94Q3 and PnUGT94Q25-V2 (with the highest enzyme activity toward substrates Rh2, Rh1 and Rd, respectively) were designated as 100%. The relative enzyme activities of the left UGT94s toward substrates Rh2, Rh1 and Rd were standardized by comparing with the three representative ones, respectively. Only bootstrap values above 70% are shown in the tree. (**C**) Tandem repeat array of *PgUGT94* homolog genes in the cloned Pg_scaffold6708 from the *P*. *ginseng* genome in our experiment, Pg_scaffold6708 assembled by Nam-Hoon Kim et al. 2018, and Scaffold8624 (partially aligned to Pg_scaffold6708) assembled by Jiang Xu et al. 2017.

In total, we obtained 26 *PgUGT94s*, including nine *PgUGT94s* already obtained from the mixed cDNA sample, from leaf and root cDNA of the individual *P*. *ginseng* plant. The deduced amino acids of the 26 *PgUGT94s* also shared high amino acid identity, ranging from 82.06% to 99.78% (Table S4). Functional assays showed that these PgUGT94s also exhibited sugar chain elongation activity at C3-O-Glc and C20-O-Glc of PPD-type, and at C6-O-Glc and C20-O-Glc of PPT-type saponins. Some of these PgUGT94s showed sugar chain elongation activity at both C3-O-Glc and C20-O-Glc of PPD-type saponins or both C3-O-Glc of PPD-type and C6-O-Glc of PPT-type as well as both C3-O-Glc of PPD-type, and C20-O-Glc of PPD- or PPT-type saponins (Fig. 2A; Fig. S1E–I; Table S3). These results suggest that PgUGT94s from an individual *P*. *ginseng* agreed with the sequence and functional diversity results obtained from a mixed cDNA sample.

The 26 PgUGT94s obtained from the individual *P*. *ginseng* plant were also classified into three clades of the phylogenetic tree, consistent with their functions and activities (Fig. 2A). Among these, half were found in clade A; most of these exhibited high substrate conversion ratios to catalyze Rh2 to produce Rg3. 12 PgUGT94s were grouped into clade C; these showed lower substrate conversion ratios to both C3-O-Glc of PPD-type and C20-O-Glc of PPD- or PPT-type saponins, or no activity. Only PgUGT94Q3 was grouped into clade B.

Some UGT94s from *Citrullus lanatus*, *Cucumis melo*, *Cucumis sativus*, and *Siraitia grosvenorii*; UGT73s from *Arabidopsis thaliana* and *Barbarea vulgaris*; and UGT720s from *C*. *lanatus* and *Siraitia* have been reported to preferentially cluster as tandem repeat arrays in their genomes^16,25^. Published *P*. *ginseng* genome data indicates that Pg_scaffold6708 (aligned with Scaffold8624 sequecned in another study^6^) and Pg_scaffold2289 harbor three and two PgUGT94 homolog sequences in tandem repeat arrays, respectively^5^. Our cloning and sequencing of a 14–26 kb fragment from Pg_scaffold6708 demonstrated that it contained three PgUGT94 homolog genes, which were identical to three detected PgUGT94 units (PgUGT94Q5-V1, PgUGT94Q11-V1, and PgUGT94Q4-V1) from the *P*. *ginseng* mixed cDNA sample, representing clades A, B, and C, respectively (Fig. 2C; Fig. S5A–C). Similarly, our cloning and sequencing of a 417.5–433 kb fragment of Pg_scaffold2289 revealed that it contained two previously detected PgUGT94 units, PgUGT94Q2 and PgUGT94Q3 (Fig. S5D and S5E).

### UGT94 diversity within a single *P. notoginseng* plant

To confirm whether UGT94 diversity is also present in *P. notoginseng*, PnUGT94 genes were cloned from leaf and root cDNA of a single *P. notoginseng* plant using five pairs of primers based on *P*. *notoginseng* transcriptome data. We obtained 313 and 210 clones from the leaf and root cDNA, respectively; these were assigned into 34 and 31 individual UGT encoding genes, respectively (Table S5). Owing to low sequence quality, seven *PnUGT94*s were excluded from further analyses. Nine *PnUGT94s*, three of which have also been cloned from a *P*. *notoginseng* leaf cDNA sample previously (Table S7), could be cloned by two or more pairs of primers or from different tissues (leaves and roots). Multiple clones were detected for 17 *PnUGT94s*, whereas only one clone was detected for the remaining 34 (Table S5). The deduced amino acid sequence of the left 51 *PnUGT94*s exhibited high amino acid identity, ranging from 84.08% to 99.78%. Functional assays showed that PnUGT94s also exhibited sugar chain elongation activity at C3-O-Glc of PPD-type saponins and C20-O-Glc of both PPD- and PPT-type saponins, as well as C6-O-Glc of PPT-type saponins, which was consistent with the sequence and functional diversity of PgUGT94s (Fig. 2B; Figs. S3 and S6).

24, 13, and 14 PnUGT94s were classified into clades A, B, and C of the phylogenetic tree, respectively (Fig. 2B). Most of the PnUGT94s in clade A exhibited higher substrate conversion ratios to catalyze sugar elongation at C3-O-Glc of PPD-type saponins, exactly like their corresponding PgUGT94s in clade A. PnUGT94s in clade B were further divided into two sub-clades; four PnUGT94s in one of these sub-clades showed sugar elongation activity at both C3-O-Glc of PPD-type and C6-O-Glc of PPT-type saponins, whereas most members of the other sub-clade showed no activity to any of the tested substrates. All of the 14 PnUGT94s in the clade C exhibited catalyzing activities at both C3-O-Glc and C20-O-Glc of PPD-type saponins, which were higher than those of their corresponding PgUGT94s in clade C. In total, seven PnUGT94s among the three clades were found to have no catalyzing activity to any tested substrates.

### Proposed biosynthetic pathways for ginsenosides with two or more sugar chain modifications

**Figure 3:**
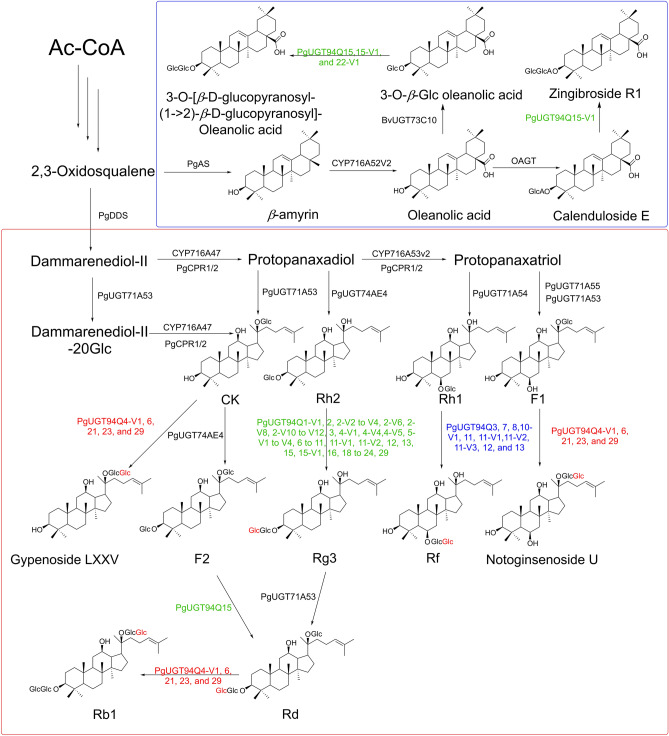
Proposed biosynthesis pathways of saponins in *Panax* plants. Ac-CoA, acetyl-CoA; PgDDS, dammarenediol synthase; PgAS, *β*-amyrin synthase; CYP, cytochrome P450; CPR, cytochrome P450 reductase; and UGT, UDP-glycosyltransferase.

Based on the cloning and functional characterization of these PgUGT94s and our previously reported PgUGT71A53, PgUGT71A54, PgUGT71A55, and PgUGT74AE4, the biosynthetic pathways of a series of ginsenosides were elucidated (Fig. 3). CK was glycosylated at C20-O-Glc by PgUGT94Q6 to form gypenoside LXXV (Figs. S2A, S7I, and S8F-I). CK was also glycosylated at C3-OH by PgUGT74AE4 to produce F2^10^, then F2 was transferred another glucose moiety at C3-O-Glc by PgUGT94Q15 to yield Rd (Fig. S2B, S7G, and S7H). Rd was converted to Rb1 by PgUGT94Q6 via transferring a glucose moiety at C20-O-Glc (Fig. 1C, S7E, S7F, and S8D). Rh2 was elongated a glucose moiety at C3-O-Glc by PgUGT94Q15 to yield Rg3 (Fig. 1A, S7A, S7B, and S8A), which could be further converted to Rd by PgUGT71A53^9^. Rh1 was elongated a glucose moiety at C6-O-Glc by PgUGT94Q3 to form Rf (Fig. 1B, S7C, S7D, S8B, and S8C). F1 could be transferred a glucose moiety at C20-O-Glc to yield notoginsenoside U (Fig. S2D, S7M, and S8E). In addition to PPD- and PPT-type saponins, UGT94s were demonstrated to be involved in the biosynthesis of oleanolic acid-type saponins. 3GOA was glycosylated at C3-O-Glc by PgUGT94Q15, Q15-V1, or Q22-V1 to produce 3-*O*-[*β*-D-glucopyranosyl-(1 → 2)-*β*-D-glucopyranosyl]-oleanolic acid (Figure S2C, S7L and S8J-O), and calunduloside E was transferred a glucose moiety at C3-O-GlcA by PgUGT94Q15-V1 to yield zingibroside R1 (Fig. S2E, S7J, and S7K). Combined with functional characterization of UGTs from *P*. *ginseng*, the possible *Panax* biosynthesis pathway net is shown in Fig. 3. Notably, the complete biosynthetic pathway of gypenoside LXXV, notoginsenoside U, Rf, Rb1, 3-*O*-[*β*-D-glucopyranosyl-(1 → 2)-*β*-D-glucopyranosyl]-oleanolic acid, and zingibroside R1 are all elucidated for the first time. These results will provide a foundation for manufacturing these ginsenosides from glucose by building microbial cell factories, and further promote the application of these ginsenosides.

In summary, the results of this study demonstrate the existence of a variety of UGT94 genes with high amino acid identity but diverse functions in an individual *P*. *ginseng* or *P*. *notoginseng* genome. The phenomenon of several UGTs sharing high amino acid identity but exhibiting diverse functions has been reported in a few plants including *A*. *thaliana* and *B*. *vulgaris*. Eight BvUGT73Cs (BvUGT73C11, BvUGT73C13, BvUGT73C21, BvUGT73C22, BvUGT73C23, BvUGT73C25, BvUGT73C26, and BvUGT73C27) from *B*. *vulgaris* were found to share high amino acid identity, ranging from 70.9% to 93.3%. The functions of these eight BvUGT73Cs were partly overlapping but diverse. BvUGT73C11, BvUGT73C21, BvUGT73C26, and BvUGT73C27 exclusively catalyzed glucosylation at C3-OH of both oleanolic acid and hederagenin. BvUGT73C13 and BvUGT73C22 exhibited glycosylation at both C3-OH and C28-OH of oleanolic acid and hederagenin. BvUGT73C23 and BvUGT73C25 exhibited glycosylation at both C3-OH and C28-OH of oleanolic acid and hederagenin, and at C23-OH of hederagenin^25^.

Some UGT94s from *Panax* plants, such as PgUGT94 Q1-V1, Q2, Q3, Q4-V1, Q4-V2, Q5-V3, Q22, Q23, and Q29 for *P*. *ginseng* and PnUGT94Q25, Q26-V17, and Q31-V7 for *P*. *notoginseng*, were relatively conservative among individuals, but most of these may tend to be polymorphic between individuals. Our results also indicate that most UGT94s tend to be expressed in different organs, since many UGT94s cloned from leaves were different from those from roots within the same *Panax* plant. However, these UGT94s shared high amino acid identity, even among UGT94s from *P*. *ginseng* and *P*. *notoginseng*, with amino acid identities ranging from 80.94% to 100% (Table S9), similar to UGT94s from a single *P*. *notoginseng* genome (84.08%–99.78%).

Most PgUGT94s, including those in clades B and C, exhibited sugar elongation activity to transfer glucose moieties to C3-O-Glc of PPD-type saponins. The phylogenetic tree implied that PgUGT94s in clade C might first branch off from clades A and B, followed by clades A and B branching off from each other (Fig. S6). Therefore, it is likely that evolution endowed an ancient PgUGT94 belonging to clade C with the ability to glycosylate C20-O-Glc of PPD-type saponins, which further developed into the ability to glycosylate C3-O-Glc of PPD-type saponins. Similarly, PgUGT94s belonging to clade B evolved the ability to glycosylate C6-O-Glc of PPT-type saponins, which further developed into the ability to glycosylate C3-O-Glc of PPD-type saponins. In this manner, more selective and efficient C3-O-Glc glycosylation developed for PPD-type saponins.

Our results confirm that some PgUGT94s, such as PgUGT94Q5-V1, PgUGT94Q11-V1, and PgUGT94Q4-V1, as well as PgUGT94Q2 and PgUGT94Q3, are closely located in tandem repeat arrays within a genome. In their genome sequencing study of *P*. *ginseng*^6^, Xu et al. proposed that the same subfamily of UGTs preferentially clustered as tandem repeat arrays within the *P*. *ginseng* genome. Recent whole-genome duplications events have suggested the duplication of subfamily UGT genes to increase their diversity. However, genome sequencing of *P*. *ginseng* and *P*. *notoginseng* has revealed few subfamily UGTs to support such a proposal; for example, only five and four (identity > 50%; coverage > 50%) of UGT94 homolog sequences were assembled, respectively^4–7^. The unprecedented diversity of UGT94s was revealed through cloning of *P*. *ginseng* and *P*. *notoginseng* cDNA, which avoided bias to complete the establishment of high-identity genes by genome sequencing. The unprecedented diversity revealed in this study may have resulted from whole-genome duplication and tandem duplication, as shown among other subfamily UGT genes of other plants. For example, UGT73C1 to UGT73C6 were discovered to be located in tandem repeat arrays on chromosome 2 of *A*. *thaliana*, at least four UGTs of UGT73Cs from *B*. *vulgaris* were organized in a tandem array on pseudomolecule 3 of *B*. *vulgaris*, and three UGTs from *S*. *grosvenorii*, UGT94-289–1, UGT94-289–2, and UGT94-289–3, were localized in tandem repeat arrays on scaffold_1277^16,25^. The organization of tandem repeat arrays likely implies gene duplication events resulting in subfunctionalization and neofunctionalization. In addition to the possible tandem repeats in genome, alleles may also contribute to the high diversity of PgUGT94s or PnUGT94s because *P. ginseng* is tetraploid and *P. notoginseng* is diploid. Further evidence is required to determine why there exist so many multi-UGT94 genes with high amino acid identity but diverse functions or activities, even within an individual *P*. *ginseng* or *P*. *notoginseng* genome.

## Experimental procedures

### Cloning of UDP-glycosyltransferases

RNA was isolated from callus, leaf, and root of *P. ginseng* as well as leaf and root of *P. notoginseng* using RNAprep Pure Plant Kit (TIANGEN, Beijing, China) according to the manufacturer's instructions. RNA was then converted into cDNA using the PrimeScript RT reagent Kit with gDNA eraser (Takara, Dalian, China). PCR amplification of the potential PgUGT94 encoding genes were performed using four pairs of primers (P29-F/R, P75-F/R, P84-F/R, and P85-F/R), and mixed cDNA of a *P. ginseng* plant and callus, cDNA of *P. ginseng* leaves, and cDNA of P. *ginseng* roots as the template, respectively. PCR amplification of the potential PnUGT94 encoding genes were performed using five pairs of primers (PN29-F/R, PN75-F/R, PN84-F/R, PN85-F/R, and PN113586-F/R), and cDNA of *P. notoginseng* leaves and cDNA of *P. notoginseng* roots as template, respectively. The PCR amplification was performed with high-fidelity PrimeSTAR HS DNA Polymerase kit. Then PCR products were cloned into the pMD18T vector (Takara, Dalian, China). The sequences of all *UGT94* genes, their expression products and related information are deposited in the Registry and Database of Bioparts for Synthetic Biology (https://www.biosino.org/npbiosys/, accession No. OENC 37 to 115, 117, 119 to 128, 130 to 141, 144 to 153, 155 to 163, 165 to 167). The official names of UGTPg1, UGTPg24, UGTPg100, UGTPg101, and all of UGT94s were designated by the UGT nomenclature committee^24^ (Table S1, S3, S5, and S7).

### Phylogenetic tree construction

The deduced amino acid sequences of UGT94s and three other UGT94 family members were aligned using clustal W in MEGA7 program (Version 7.0.26) with default parameters. The MEGA7 program was used to construct the Neighbor–Joining phylogenetic tree with set parameters (bootstrap method with 1,000 bootstrap replicates, poisson model, uniform rates and complete deletion). The scale bar means 0.1 amino acid substitution per site. The percentage of bootstrap values were marked near the nodes as numbers.

### Heterologous expression of the UGT94 genes

Heterologous expression of the UGT94 genes in *E. coli* BL21 (DE3) was performed as described previously with slight modifications^11^. UGT94 genes with a C-terminal 6 × His-tag were constructed into the pET28a vector using the ClonExpress Multis one Step Cloning Kit (Vazyme Biotech) and transformed into *E. coli* BL21 (DE3). After induction with 0.2 mM isopropyl β-D-1-thiogalactopyranoside, the cells were collected by centrifugation at 8,000 g for 5 min, and suspended with 50 mM Tris–HCl buffer (pH 8.0). The cells were disrupted with a French Press at 25 kpsi. The homogenate was centrifuged at 12, 000 g for 20 min to remove cell debris and the left supernatant was used for in vitro enzymatic assays and western blotting. The *E. coli* BL21 (DE3) cells harboring the empty vector pET28a were carried out in parallel as the control.

### Enzymatic assays for UGT94s

Enzymatic assays for UGT94s were carried out as described by Yan et al.^9^ The in vitro enzymatic reaction was conducted in a 50 μl volume containing 50 mM Tris–HCl buffer (pH 8.0), 1% Tween-20, 5 mM UDP-glucose, 0.5 mM acceptor substrate, and 40 ul crude enzyme (the recombinant *E. coli* extract) in a 35 °C water bath for 12 h. The reaction was terminated by adding 50 μl of *n*-butanol. The *n*-butanol phase was evaporated and then analyzed by thin layer chromatography (TLC) and high-performance liquid chromatography (HPLC). The concentration of each His-tagged UGT94 in crude enzyme was quantified by dot blot analysis as previous described^26^. A series of diluted (8, 12, 14, 16, 18, and 32 ng/μL) of purified C-terminal 6× His-tagged UGT OleD (NCBI accession number, ABA42119.2) were used to draw a standard curve for protein quantitative. To compare the enzymatic activities of UGT94s, the enzymatic reaction condition was the same as describe above except the incubation time was 1 h. The enzyme activity of each UGT94 was calculated as the amount of substrate (nM) converted by per unit protein (mg) in 1 min, and all data are means of three independent experiments (Table S11). The relative enzyme activities of three representative UGT94s with the highest enzyme activity toward substrate Rh2 (C3), Rh1 (C6) and Rd (C20) respectively, were designated as 100%. The relative enzyme activities of each of the left UGT94s toward Rh2, Rh1 and Rd were standardized by comparing with the three representative ones, PgUGT94Q18, PgUGT94Q3 and PnUGT94Q25-V2, respectively.

### Cloning of Pg_scaffold6708 and Pg_scaffold2289

The 14–26 kb of Pg_scaffold6708 was divided in five parts. The each parts were cloned using the genome DNA of *P. ginseng* callus as the template and linked into pEASY-Blunt Simple cloning Vector (Transgen, Beijing, China) to sequence. The 417.5–419.9 kb and 430.2–433 kb of Pg_scaffold2289 were PCR amplified using the genome DNA of *P. ginseng* callus as template and also linked into the pEASY-Blunt vector to sequence.

### TLC and HPLC analysis

TLC analysis was performed using silica gel 60 F254 plates (Merck KGaA, Darmstadt, Germany) as described previously^11^. Ginsenoside standards were purchased from Nantong FeiYu biological technology Co., Ltd. (Nantong, China). The concentration of ginsenoside standards is 1 mg/ml each. HPLC analysis was performed on a Shimadzu LC 20A system (Shimadzu, Kyoto, Japan). Chromatographic separations were performed with a Shim-pack XR-ODS column (100 mm × 2.0 mm, 2.2 μm, Shimadzu, Kyoto, Japan). Water (A) and acetonitrile (B) were used in the gradient elution system. The HPLC analysis program for reactants was carried out as follows: 0–2 min (15% B), 16 min (70% B), and 16.01–20 min (95% B), and 20.01–22 min (15% B). The flow rate was kept at 0.45 ml/min and the temperature of column compartment was set as 35℃. The products were detected at 203 nm.

### HPLC/ESIMS analysis

The chromatographic analysis was carried out on a Waters LC system (Waters, Massachusetts, USA) using a Shim-pack XR-ODS column (100 mm × 2.0 mm, 2.2 μm, Shimadzu, Kyoto, Japan). The HR-ESIMS data were acquired using a Thermo Scientific Q Exactive mass spectrometer (Thermo Fisher Scientific, Massachusetts, USA) in the positive or negative ionization mode.

### NMR analysis

^1^H NMR, ^13^C NMR, 2D-COSY NMR, 2D-NOE NMR, 2D-HSQC NMR and 2D-HMBC NMR were recorded on Bruker AM–500 (500 MHz) spectrometers in the C_5_D_5_N or d6-DMSO.

Ginsenoside Rg3

^1^H NMR (C_6_H_5_N, 500 MHz) δ ppm: 5.38 (d, *J* = 7.6 Hz, 1H), 5.31 (t, *J* = 7.0 Hz, 1H), 4.56 (d, *J* = 11.6 Hz, 1H), 4.52–4.44 (m, 2H), 4.39–4.29 (m, 3H), 4.28–4.21 (m, 2H), 4.19–4.11 (m, 2H), 3.95–3.84 (m, 3H), 3.29 (dd, *J* = 11.8, 4.4 Hz, 1H), 2.67–2.55 (m, 1H), 2.41–2.24 (m, 2H), 2.22–2.15 (m, 1H), 2.09–1.98 (m, 3H), 1.96–1.76 (m, 2H), 1.75–1.67 (m, 1H), 1.65 (s, 3H), 1.62 (s, 3H), 1.60–1.44 (m, 5H), 1.43 (s, 3H), 1.42–1.32 (m, 3H), 1.29 (s, 3H), 1.24–1.18 (m, 1H), 1.11 (s, 3H), 1.07–1.00 (m, 1H), 0.97 (s, 3H), 0.80 (s, 3H), 0.78–0.70 (m, 1H), 0.68 (d, *J* = 11.2 Hz, 1H).

Ginsenoside Rf

^1^H NMR (C_6_H_5_N, 500 MHz) δ ppm: 5.93 (d, *J* = 7.6 Hz, 1H), 5.38–5.30 (m, 1H), 4.95–4.90 (m, 1H), 4.53–4.43 (m, 3H), 4.40–4.06(m, 8H), 3.98–3.82 (m, 3H), 3.50–3.44 (m, 1H), 2.65–2.55 (m, 1H), 2.45–2.40 (m, 1H), 2.35–2.25 (m, 2H), 2.14–1.99 (m, 6H), 1.98–1.76 (m, 5H), 1.72–1.60 (m, 8H), 1.55–1.50 (m, 2H), 1.47 (s, 3H), 1.43–1.29 (m, 5H), 1.20–1.12 (m, 4H), 1.01–0.91 (m, 4H), 0.80 (s, 3H);

^13^C NMR (C_6_H_5_N, 125 MHz) δ ppm: 130.70, 126.27, 103.85, 103.78, 79.82, 79.66, 78.56, 78.37, 78.02, 77.79, 75.98, 72.90, 71.67, 70.95, 63.29, 61.36, 54.71, 51.61, 50.80, 48.22, 44.97, 41.09, 40.14, 39.58, 39.37, 35.75, 32.01, 31.18, 27.70, 26.96, 26.76, 25.74, 22.93, 17.61, 17.53, 17.35, 16.74, 16.68.

Ginsenoside Rb1

^1^H NMR (C_6_H_5_N, 500 MHz) δ ppm: 5.36 (d, *J* = 7.6 Hz, 1H), 5.32–5.27 (m, 1H), 5.13–5.06 (m, 2H), 4.90 (d, *J* = 7.6 Hz, 1H), 4.71 (d, *J* = 11.2 Hz, 1H), 4.58–4.43 (m, 4H), 4.36–4.00 (m, 14H), 3.25 (dd, *J* = 11.6, 4.0 Hz, 1H), 2.64–2.28 (m, 4H), 2.21–2.06 (m, 1H), 2.01–1.90 (m, 2H), 1.88–1.73 (m, 3H), 1.64 (s, 6H), 1.59 (m, 3H), 1.57–1.40 (m, 4H), 1.38–1.14 (m, 11H), 1.09 (s, 3H), 1.02–0.96 (m, 1H), 0.95 (s, 6H), 0.80 (s, 3H), 0.75–0.60 (m, 2H).

Notoginsenoside U

^1^H NMR (d6-DMSO, 500 MHz) δ ppm: 5.07 (t, *J* = 6.5 Hz, 1H), 4.44 (d, *J* = 7.4 Hz, 1H), 4.24 (d, *J* = 7.6 Hz, 1H), 3.94–3.84 (m, 2H), 3.68–3.62 (m, 1H), 3.59–3.53 (m, 1H), 3.43–3.28 (m, 2H), 3.21–3.08 (m, 2H), 3.08–3.00 (m, 3H), 2.22–2.13 (m, 1H), 2.06–1.86 (m, 3H), 1.80–1.66 (m, 2H), 1.63 (s, 3H), 1.65–1.58 (m, 1H), 1.56 (s, 3H), 1.54–1.34 (m, 6H), 1.26–1.19 (m, 8H), 1.08–1.00 (m, 1H), 0.94 (s, 3H), 0.95–0.87 (m, 1H), 0.83 (s, 9H), 0.74 (d, *J* = 10.2 Hz, 1H).

Gypenoside LXXV

^1^H NMR (C_6_H_5_N, 500 MHz) δ ppm: 5.35–5.29 (m, 1H), 5.12 (d, *J* = 7.8 Hz, 1H), 5.09 (d, *J* = 7.8 Hz, 1H), 4.73 (d, *J* = 11.4 Hz, 1H), 4,52 (d, *J* = 11.8 Hz, 1H), 4.39–4.28 (m, 2H), 4.26–4.12 (m, 3H), 4.10–3.99 (m, 3H), 3.98–3.87 (m, 2H), 3.76–3.62 (m, 1H), 3.44–3.37 (m, 1H), 2.66–2.23 (m, 2H), 2.47–2.32 (m, 2H), 2.08–1.97 (m, 2H), 1.90–1.75 (m, 4H), 1.66 (s, 6H), 1.71–1.65 (m, 1H), 1.60 (s, 3H), 1.59–1.30 (m, 7H), 1.26–1.23 (m, 1H), 1.22 (s, 3H), 1.03 (s, 3H), 1.02–0.97 (m, 1H), 0.99 (s, 3H), 0.94 (s, 3H), 0.91–0.85 (m, 1H), 0.88 (s, 3H), 0.80 (d, *J* = 11.4 Hz, 1H);

^13^C NMR (C_6_H_5_N, 125 MHz) δ ppm: 130.99, 125.90, 105.31, 98.01, 83.39, 79.21, 78.33, 78.27, 77.98, 76.99, 75.18, 74.78, 71.62, 71.49, 70.16, 70.08, 62.73, 56.28, 51.54, 51.32, 50.24, 49.39, 39.99, 39.48, 39.32, 37.28, 36.14, 35.09, 30.78, 30.66, 28.60, 28.18, 26.55, 25.73, 23.12, 22.29, 18.68, 17.89, 17.35, 16.27, 16.25, 15.98.

3-*O*-[*β*-D-glucopyranosyl-(1 → 2)-*β*-D-glucopyranosyl]-oleanolic acid:

^1^H NMR (C_6_H_5_N, 500 MHz) δ ppm: 5.46 (brs, 1H), 5.37–5.33 (m, 1H), 4.90 (dd, *J* = 7.3, 2.2 Hz, 1H), 4.53 (d, *J* = 12.0 Hz, 1H), 4.49–4.41 (m, 2H), 4.39–4.27 (m, 3H), 4.25–4.19 (m, 2H), 4.18–4.07 (m, 2H), 3.99–3.87 (m, 2H), 3.31–3.24(m, 2H), 2.25–1.69 (m, 1H), 1.50–1.36 (m, 4H), 1.28 (s, 6H), 1.25–1.15 (m, 4H), 1.08 (s, 3H), 1.00 (s, 3H), 0.97 (s, 3H), 0.94 (s, 3H), 0.88–0.83 (m, 1H), 0.80 (s, 3H), 0.71 (d, *J* = 11.6 Hz, 1H);

^13^C NMR (C_6_H_5_N, 125 MHz) δ ppm: 179.97, 144.65, 122.28, 105.79, 104.81, 88.74, 83.17, 77.99, 77.78, 77.68, 76.88, 71.40, 71.35, 62.57, 62.44, 55.59, 47.75, 46.43, 46.26, 41.92, 41.76, 39.49, 39.27, 38.47, 36.71, 34.00, 33.66, 32.97, 30.73, 28.07, 27.98, 26.34, 25.95, 23.46, 18.23, 17.15, 16.59, 15.23.

## Supplementary information

Supplementary Table

Supplementary Figure
