# Supplementary material for: The unprecedented diversity of UGT94-family UDP-glycosyltransferases in Panax plants and their contribution to ginsenoside biosynthesis
Source: Sci Rep. 2020 Sep 21;10:15394. doi: 10.1038/s41598-020-72278-y (PMC7506552; doi:10.1038/s41598-020-72278-y)
Supplement: Supplementary file 2 — Supplementary Figure [file 41598_2020_72278_MOESM2_ESM.pdf]

Supplementary Information

The unprecedented diversity of UGT94-family UDP-glycosyltransferases in *Panax* plants and their contribution to ginsenoside biosynthesis

Chengshuai Yang[1, 2, 5], Chaojing Li[1, 4, 5], Wei Wei[1, 5], Yongjun Wei[1], Qunfang Liu[3], Guoping Zhao[1, 2], Jianmin Yue[3], Xing Yan[1, *], Pingping Wang[1, *], Zhihua Zhou[1, *]

[1]CAS-Key Laboratory of Synthetic Biology, CAS Center for Excellence in Molecular Plant Sciences, Institute of Plant Physiology and Ecology, Shanghai Institutes for Biological Sciences, Chinese Academy of Sciences, Shanghai 200032, China

[2]Institute of Synthetic Biology, Shenzhen Institutes of Advanced Technology, Chinese Academy of Sciences, Shenzhen 518055, China

[3]State Key Laboratory of Drug Research, Shanghai Institute of Materia Medica, Chinese Academy of Sciences, Shanghai 201203, China

[4]University of Chinese Academy of Sciences, Beijing 100049, China

[5]These authors contributed equally to this work.

[*]Correspondence authors.

E-mail addresses: Zhihua Zhou (zhouzhihua@sippe.ac.cn), Pingping Wang (ppwang@sibs.ac.cn), Xing Yan (yanxing@sibs.ac.cn).

Tel: Zhihua Zhou (86-21-549240450), Pingping Wang (86-21-54924049), Xing Yan (86-21-54924049).

Fax: Zhihua Zhou (86-21-54924049), Pingping Wang (86-21-54924049), Xing Yan (86-21-54924049).

Figure S1. TLC analysis of the activities of the PgUGT94s toward Rh2, Rh1, Rd, and CK with UDP-glucose as sugar donor.

(A) TLC analysis of the activities of the PgUGT94s from mixed cDNA of a *P. ginseng* plant and callus toward Rh2 with UDP-glucose as sugar donor.

(B) TLC analysis of the activities of the PgUGT94s from mixed cDNA of a *P. ginseng* plant and callus toward Rh1 with UDP-glucose as sugar donor.

(C) TLC analysis of the activities of the PgUGT94s from mixed cDNA of a *P. ginseng* plant and callus toward Rd with UDP-glucose as sugar donor.

(D) TLC analysis of the activities of the PgUGT94s from mixed cDNA of a *P. ginseng* plant and callus toward CK with UDP-glucose as sugar donor.

(E) TLC analysis of the activities of the PgUGT94s from an individual *P. ginseng* plant toward Rh2 with UDP-glucose as sugar donor.

(F) TLC analysis of the activities of the PgUGT94s from an individual *P. ginseng* plant toward Rh1 with UDP-glucose as sugar donor.

(G) TLC analysis of the activities of the PgUGT94s from an individual *P. ginseng* plant toward Rd with UDP-glucose as sugar donor.

(H) TLC analysis of the activities of the PgUGT94s from mixed cDNA of a *P. ginseng* plant and callus toward F1 with UDP-glucose as sugar donor.

(I) TLC analysis of the activities of the PgUGT94s from mixed cDNA of a *P. ginseng* plant and callus toward calunduloside E with UDP-glucose as sugar donor.

A

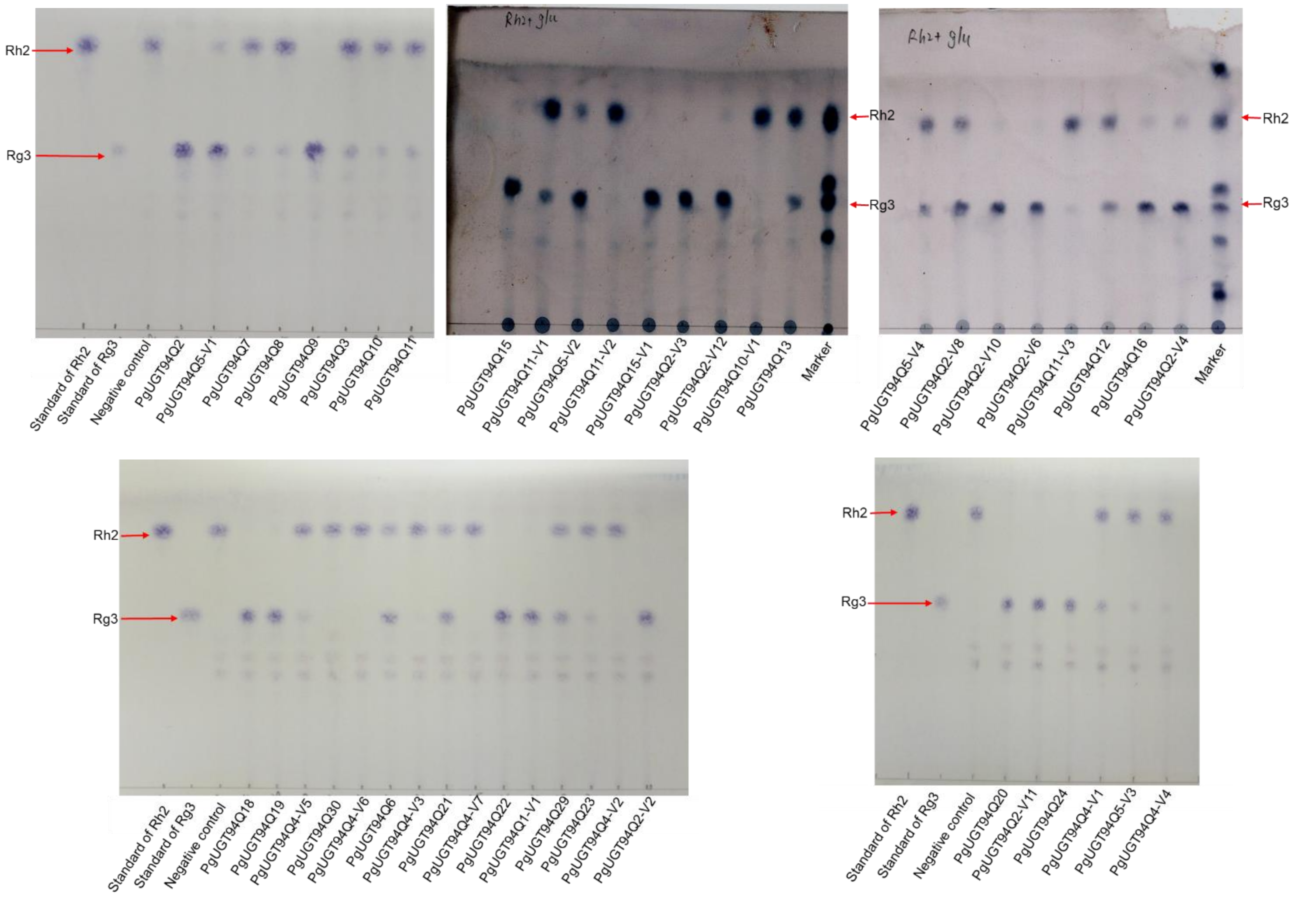

B

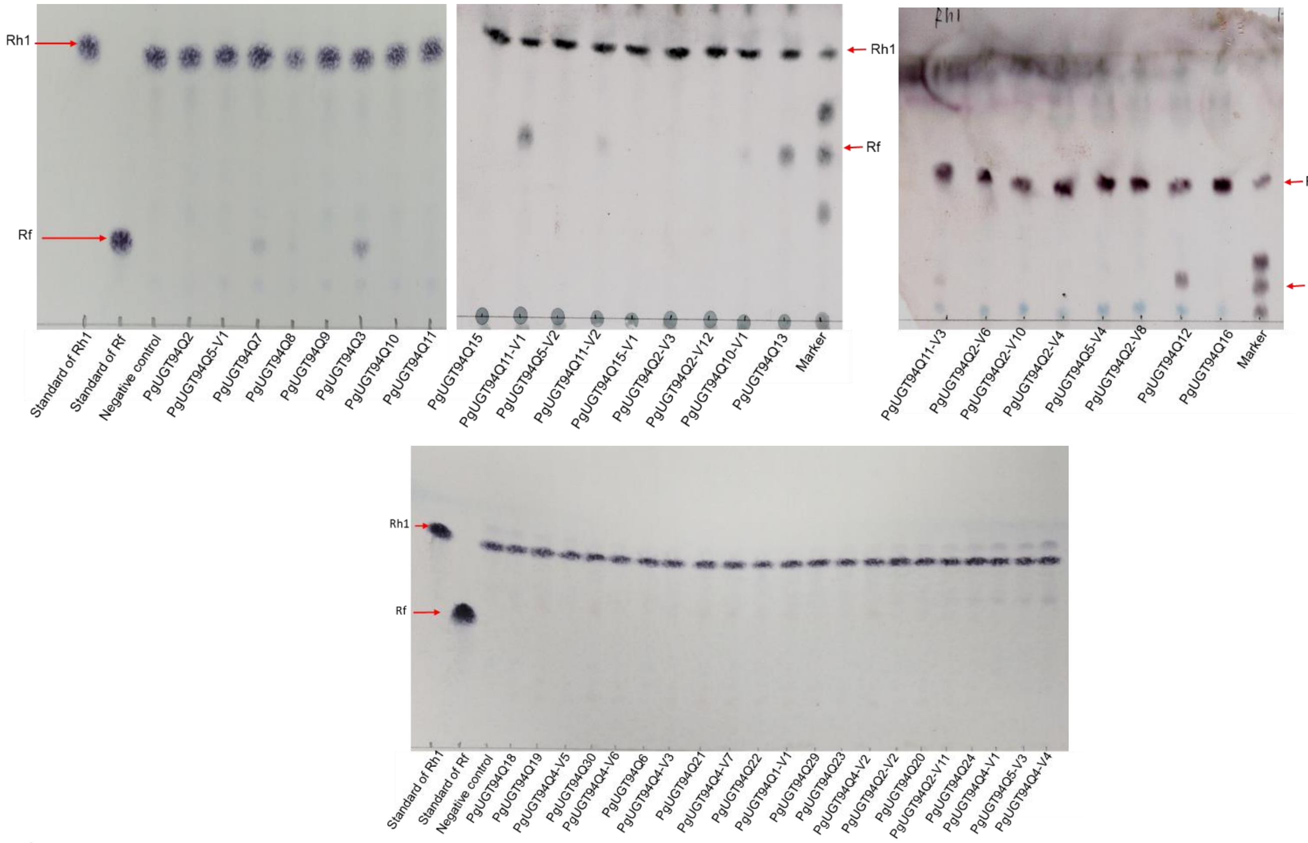
Rh1
Rf
Standard of Rh1
Standard of Rf
Negative control
PgUGT94Q2
PgUGT94Q5-V1
PgUGT94Q7
PgUGT94Q8
PgUGT94Q9
PgUGT94Q3
PgUGT94Q10
PgUGT94Q11
PgUGT94Q15
PgUGT94Q11-V1
PgUGT94Q5-V2
PgUGT94Q11-V2
PgUGT94Q15-V1
PgUGT94Q2-V3
PgUGT94Q2-V12
PgUGT94Q10-V1
PgUGT94Q13
Marker
PgUGT94Q11-V3
PgUGT94Q2-V6
PgUGT94Q2-V10
PgUGT94Q2-V4
PgUGT94Q5-V4
PgUGT94Q2-V8
PgUGT94Q12
PgUGT94Q16
Marker
Standard of Rh1
Standard of Rf
Negative control
PgUGT94Q18
PgUGT94Q19
PgUGT94Q4-V5
PgUGT94Q30
PgUGT94Q4-V6
PgUGT94Q6
PgUGT94Q4-V3
PgUGT94Q21
PgUGT94Q4-V7
PgUGT94Q22
PgUGT94Q1-V1
PgUGT94Q29
PgUGT94Q23
PgUGT94Q4-V2
PgUGT94Q2-V2
PgUGT94Q20
PgUGT94Q2-V11
PgUGT94Q24
PgUGT94Q4-V1
PgUGT94Q5-V3
PgUGT94Q4-V4

C

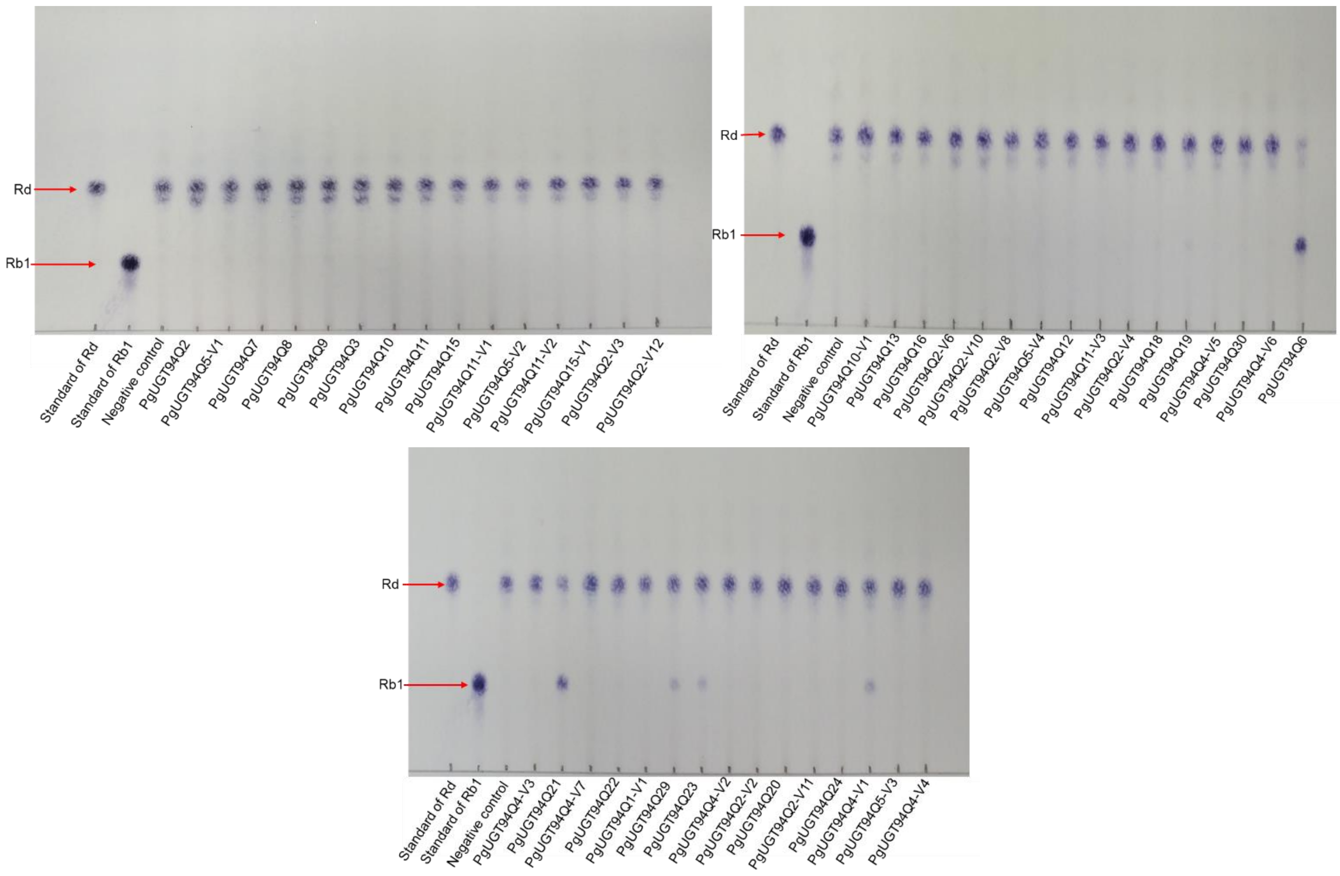
Rd
Rb1
Standard of Rd
Standard of Rb1
Negative control
PgUGT94Q2
PgUGT94Q5-V1
PgUGT94Q7
PgUGT94Q8
PgUGT94Q9
PgUGT94Q3
PgUGT94Q10
PgUGT94Q11
PgUGT94Q15
PgUGT94Q11-V1
PgUGT94Q5-V2
PgUGT94Q11-V2
PgUGT94Q15-V1
PgUGT94Q2-V3
PgUGT94Q2-V12
Standard of Rd
Standard of Rb1
Negative control
PgUGT94Q10-V1
PgUGT94Q13
PgUGT94Q16
PgUGT94Q2-V6
PgUGT94Q2-V10
PgUGT94Q2-V8
PgUGT94Q5-V4
PgUGT94Q12
PgUGT94Q11-V3
PgUGT94Q2-V4
PgUGT94Q18
PgUGT94Q19
PgUGT94Q4-V5
PgUGT94Q30
PgUGT94Q4-V6
PgUGT94Q6
Standard of Rd
Standard of Rb1
Negative control
PgUGT94Q4-V3
PgUGT94Q21
PgUGT94Q4-V7
PgUGT94Q22
PgUGT94Q1-V1
PgUGT94Q29
PgUGT94Q23
PgUGT94Q4-V2
PgUGT94Q2-V2
PgUGT94Q20
PgUGT94Q2-V11
PgUGT94Q24
PgUGT94Q4-V1
PgUGT94Q5-V3
PgUGT94Q4-V4

D

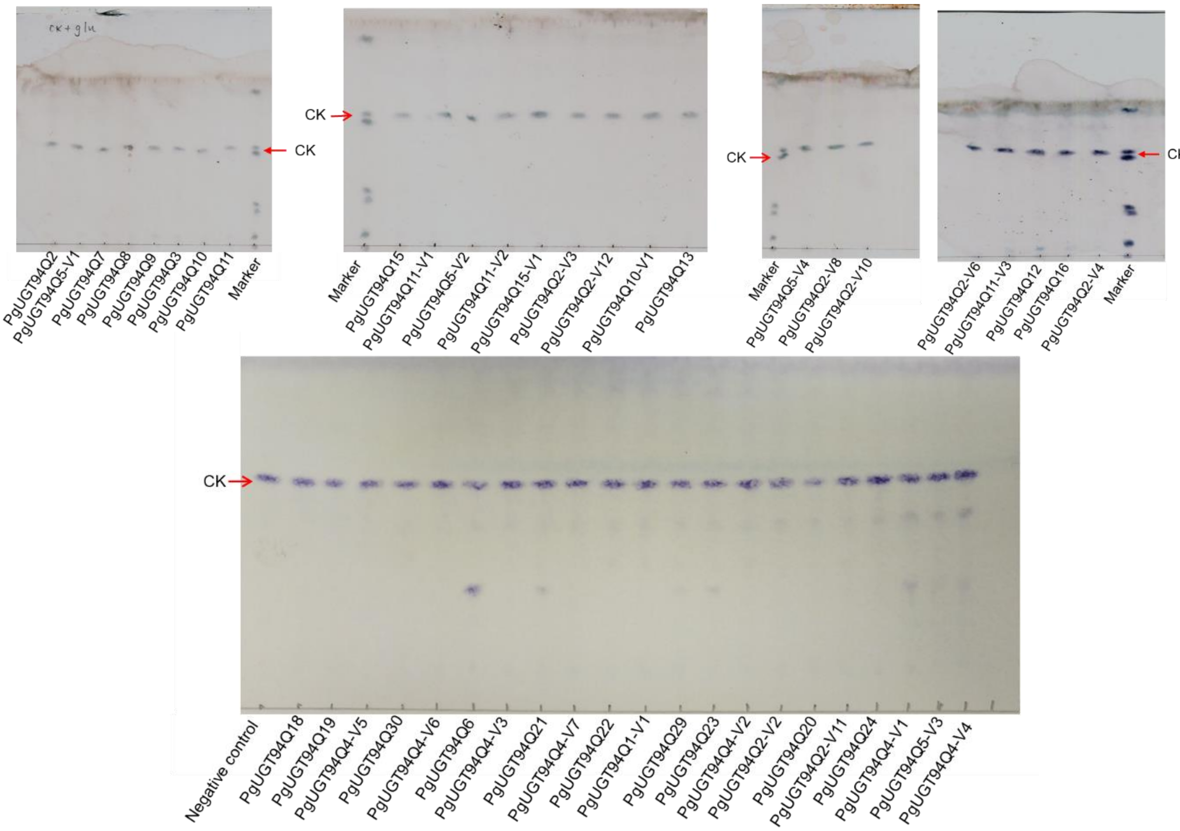
ck+glu
CK
CK
CK
CK
CK
PgUGT94Q2
PgUGT94Q5-V1
PgUGT94Q7
PgUGT94Q8
PgUGT94Q9
PgUGT94Q3
PgUGT94Q10
PgUGT94Q11
Marker
Marker
PgUGT94Q15
PgUGT94Q11-V1
PgUGT94Q5-V2
PgUGT94Q11-V2
PgUGT94Q15-V1
PgUGT94Q2-V3
PgUGT94Q2-V12
PgUGT94Q10-V1
PgUGT94Q13
Marker
PgUGT94Q5-V4
PgUGT94Q2-V8
PgUGT94Q2-V10
PgUGT94Q2-V6
PgUGT94Q11-V3
PgUGT94Q12
PgUGT94Q16
PgUGT94Q2-V4
Marker
Negative control
PgUGT94Q18
PgUGT94Q19
PgUGT94Q4-V5
PgUGT94Q30
PgUGT94Q4-V6
PgUGT94Q6
PgUGT94Q4-V3
PgUGT94Q21
PgUGT94Q4-V7
PgUGT94Q22
PgUGT94Q1-V1
PgUGT94Q29
PgUGT94Q23
PgUGT94Q4-V2
PgUGT94Q2-V2
PgUGT94Q20
PgUGT94Q2-V11
PgUGT94Q24
PgUGT94Q4-V1
PgUGT94Q5-V3
PgUGT94Q4-V4

E

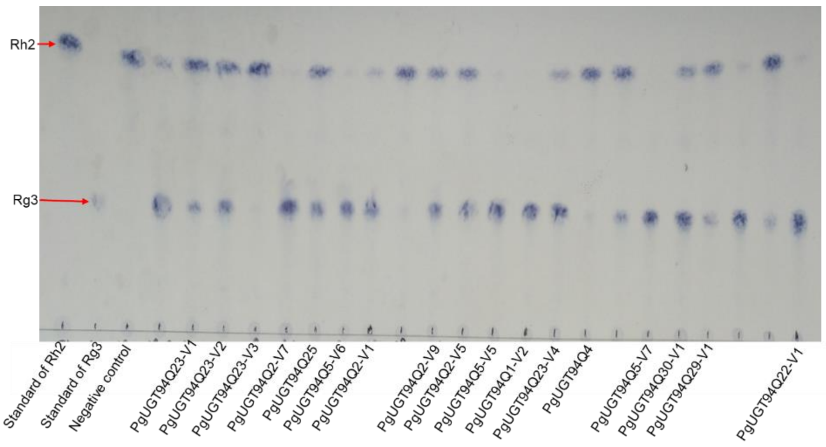
Rh2
Rg3
Standard of Rh2
Standard of Rg3
Negative control
PgUGT94Q23-V1
PgUGT94Q23-V2
PgUGT94Q23-V3
PgUGT94Q2-V7
PgUGT94Q25
PgUGT94Q5-V6
PgUGT94Q2-V1
PgUGT94Q2-V9
PgUGT94Q2-V5
PgUGT94Q5-V5
PgUGT94Q1-V2
PgUGT94Q23-V4
PgUGT94Q4
PgUGT94Q5-V7
PgUGT94Q30-V1
PgUGT94Q29-V1
PgUGT94Q22-V1

F

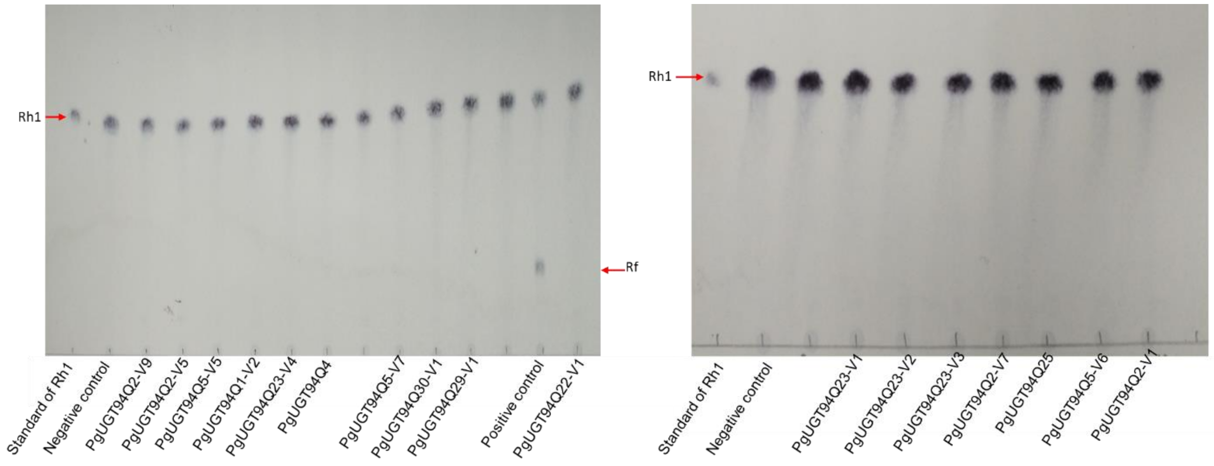
Rh1
Rf
Standard of Rh1
Negative control
PgUGT94Q2-V9
PgUGT94Q2-V5
PgUGT94Q5-V5
PgUGT94Q1-V2
PgUGT94Q23-V4
PgUGT94Q4
PgUGT94Q5-V7
PgUGT94Q30-V1
PgUGT94Q29-V1
Positive control
PgUGT94Q22-V1
Rh1
Standard of Rh1
Negative control
PgUGT94Q23-V1
PgUGT94Q23-V2
PgUGT94Q23-V3
PgUGT94Q2-V7
PgUGT94Q25
PgUGT94Q5-V6
PgUGT94Q2-V1

G

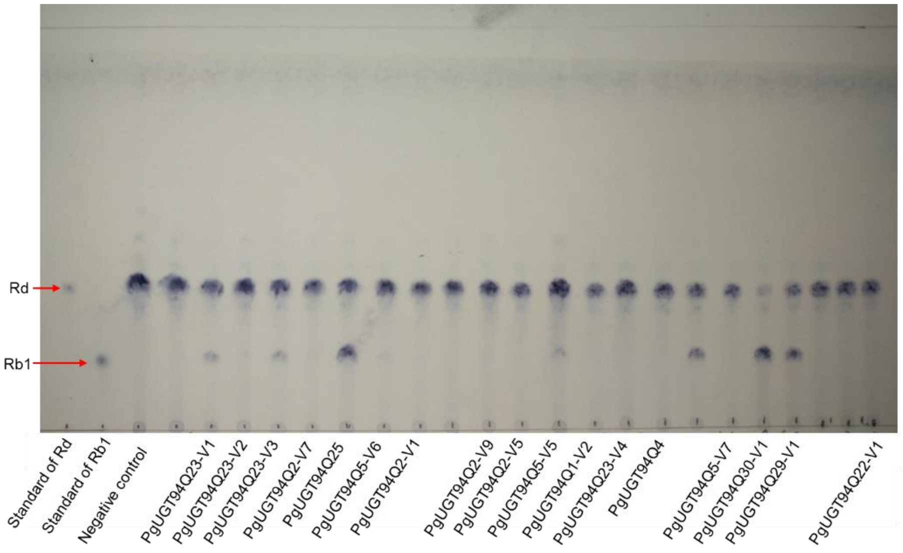

Rd
Rb1
Standard of Rd
Standard of Rb1
Negative control
PgUGT94Q23-V1
PgUGT94Q23-V2
PgUGT94Q23-V3
PgUGT94Q2-V7
PgUGT94Q25
PgUGT94Q5-V6
PgUGT94Q2-V1
PgUGT94Q2-V9
PgUGT94Q2-V5
PgUGT94Q5-V5
PgUGT94Q1-V2
PgUGT94Q23-V4
PgUGT94Q4
PgUGT94Q5-V7
PgUGT94Q30-V1
PgUGT94Q29-V1
PgUGT94Q22-V1

H

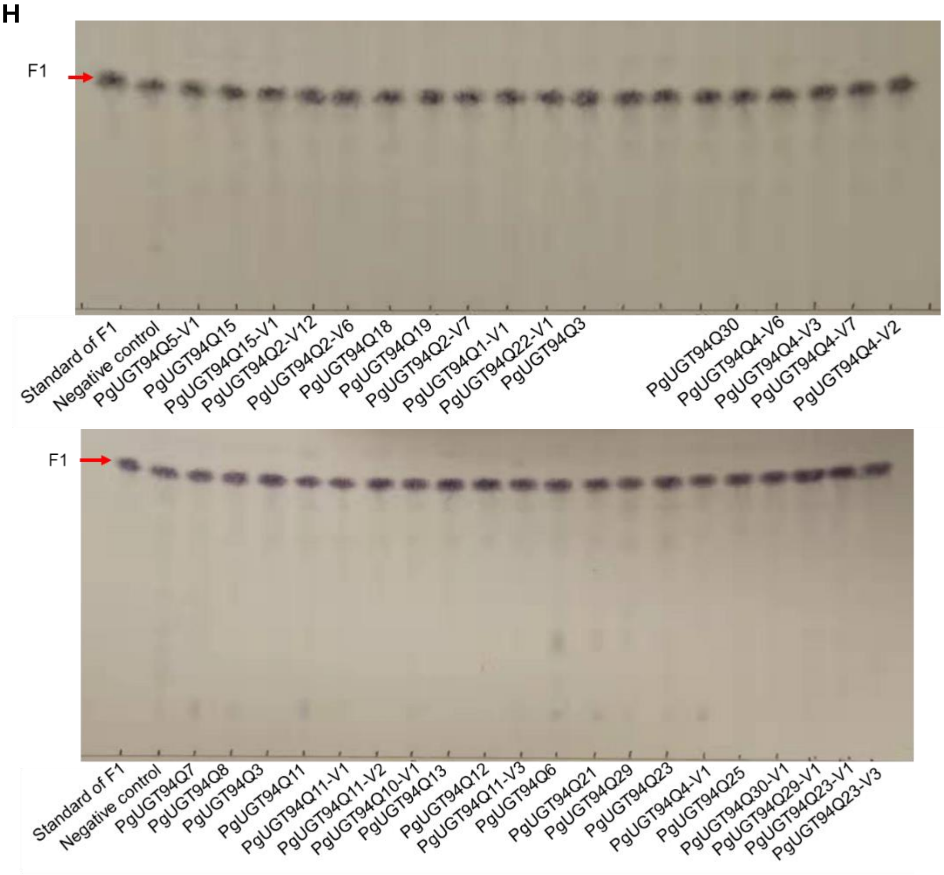

F1
Standard of F1
Negative control
PgUGT94Q5-V1
PgUGT94Q15
PgUGT94Q15-V1
PgUGT94Q2-V12
PgUGT94Q2-V6
PgUGT94Q18
PgUGT94Q19
PgUGT94Q2-V7
PgUGT94Q1-V1
PgUGT94Q22-V1
PgUGT94Q3
PgUGT94Q30
PgUGT94Q4-V6
PgUGT94Q4-V3
PgUGT94Q4-V7
PgUGT94Q4-V2
F1
Standard of F1
Negative control
PgUGT94Q7
PgUGT94Q8
PgUGT94Q3
PgUGT94Q11
PgUGT94Q11-V1
PgUGT94Q11-V2
PgUGT94Q10-V1
PgUGT94Q13
PgUGT94Q12
PgUGT94Q11-V3
PgUGT94Q6
PgUGT94Q21
PgUGT94Q29
PgUGT94Q23
PgUGT94Q4-V1
PgUGT94Q25
PgUGT94Q30-V1
PgUGT94Q29-V1
PgUGT94Q23-V1
PgUGT94Q23-V3

I

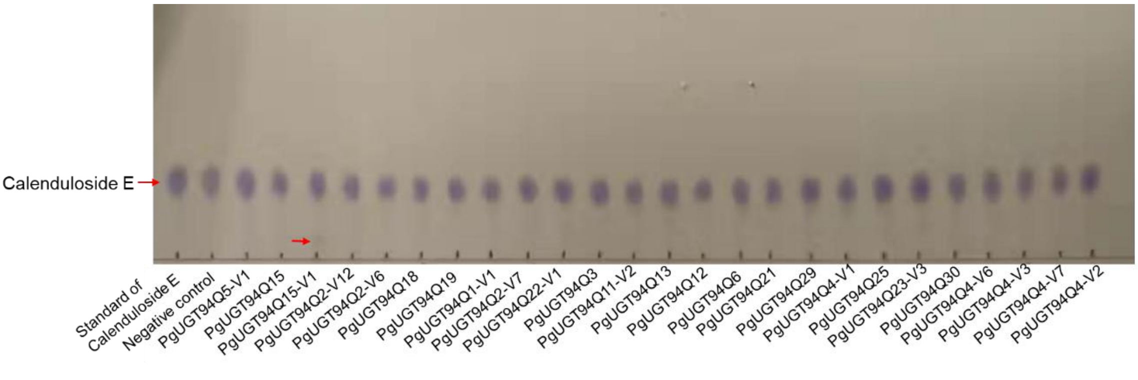

Calenduloside E
Standard of Calenduloside E
Negative control
PgUGT94Q5-V1
PgUGT94Q15
PgUGT94Q15-V1
PgUGT94Q2-V12
PgUGT94Q2-V6
PgUGT94Q18
PgUGT94Q19
PgUGT94Q1-V1
PgUGT94Q2-V7
PgUGT94Q22-V1
PgUGT94Q3
PgUGT94Q11-V2
PgUGT94Q13
PgUGT94Q12
PgUGT94Q6
PgUGT94Q21
PgUGT94Q29
PgUGT94Q4-V1
PgUGT94Q25
PgUGT94Q23-V3
PgUGT94Q30
PgUGT94Q4-V6
PgUGT94Q4-V3
PgUGT94Q4-V7
PgUGT94Q4-V2

Figure S2. Functional characterization of PgUGT94s.

(A) HPLC analysis *in vitro* reaction of PgUGT94Q6 toward CK with UDP-glucose as sugar donor.

(B) HPLC analysis *in vitro* reaction of PgUGT94Q15 toward F2 with UDP-glucose as sugar donor.

(C) HPLC analysis in vitro reactions of PgUGT94Q15, PgUGT94Q15-V1, and PgUGT94Q22-V1 toward 3-O-β-Glc oleanolic acid (3GOA) with UDP-glucose as sugar donor. The 3GOA was obtained by incubating the UGT73C10 crude enzyme with oleanolic acid and UDP-glucose, without purification. The peak near 12.5 min may be a endogenous metabolites of *E. coli* expressing UGT73C10 or PgUGT94s. The crude enzyme of empty vector pET28a was chosen as negative control. The peaks in blue frame are 3GOA. The peaks in red frame are 3-*O*-[β-D-glucopyranosyl-(1→2)-β-D-glucopyranosyl]-oleanolic acid.

(D) HPLC analysis *in vitro* reactions of PgUGT94Q6, PgUGT94Q21, PgUGT94Q29, PgUGT94Q23, and PgUGT94Q4-V1 toward F1 with UDP-glucose as sugar donor.

(E) HPLC analysis *in vitro* reactions of PgUGT94Q15-V1 toward calenduloside E with UDP-glucose as sugar donor.

A

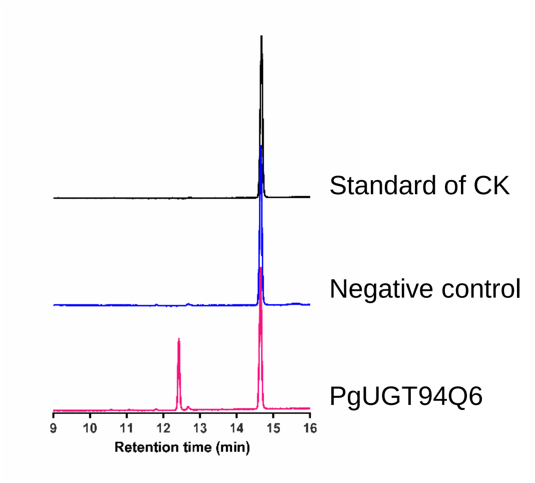

B

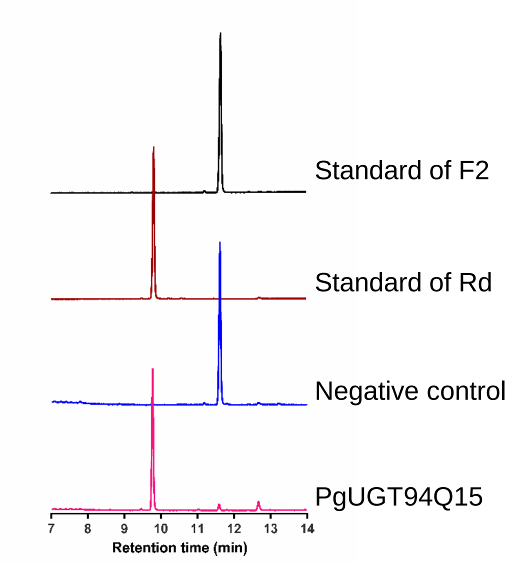

C

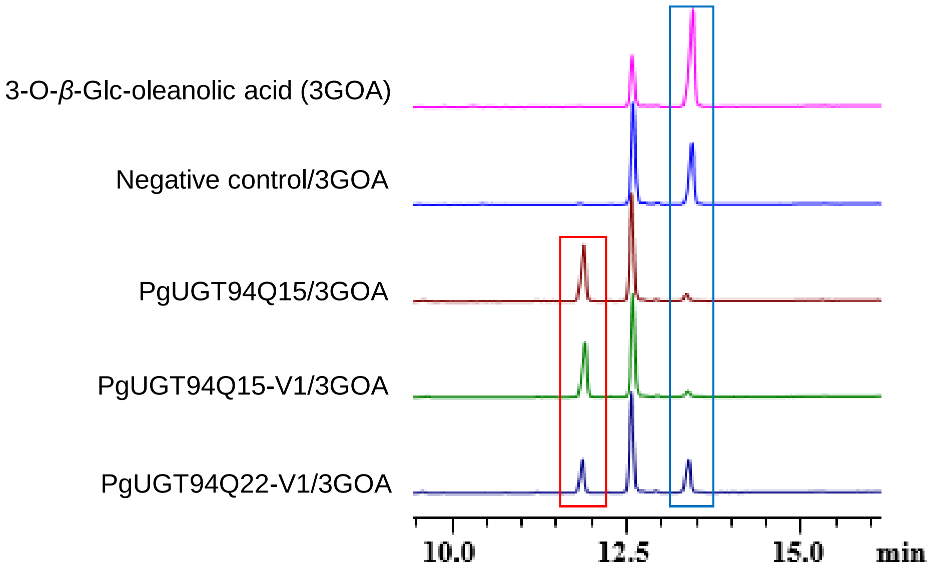

D

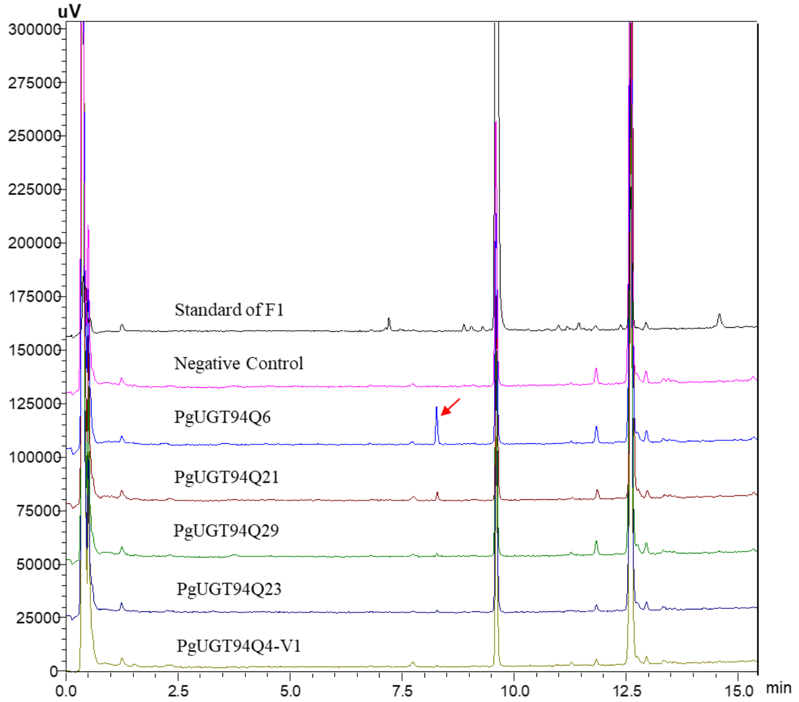
uV
300000
275000
250000
225000
200000
175000
150000
125000
100000
75000
50000
25000
0
0.0
2.5
5.0
7.5
10.0
12.5
15.0
min
Standard of F1
Negative Control
PgUGT94Q6
PgUGT94Q21
PgUGT94Q29
PgUGT94Q23
PgUGT94Q4-V1

E

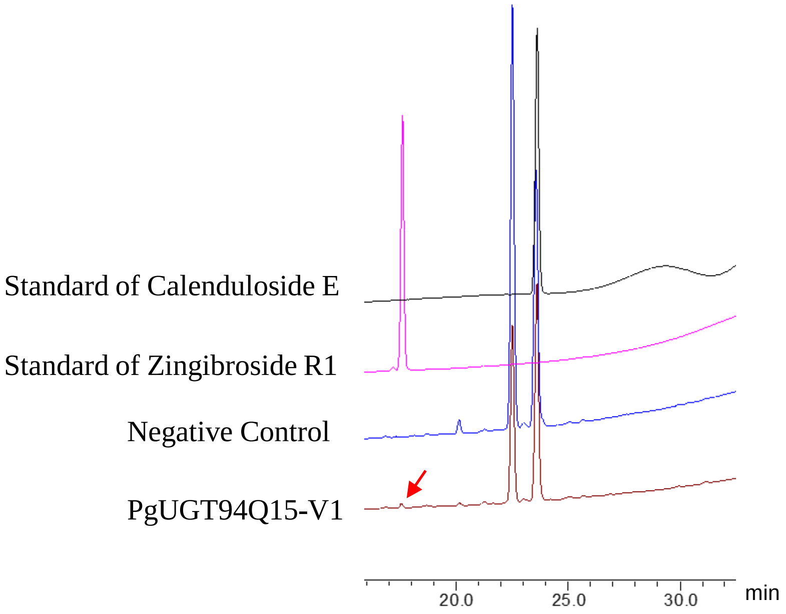
Standard of Calenduloside E
Standard of Zingibroside R1
Negative Control
PgUGT94Q15-V1
20.0
25.0
30.0
min

Figure S3. TLC analysis of the activities of the PnUGT94s toward Rh2, Rh1, Rd, and CK with UDP-glucose as sugar donor.

(A) TLC analysis of the activities of the PnUGT94s from an individual *P. notoginseng* plant toward Rh2 with UDP-glucose as sugar donor.

(B) TLC analysis of the activities of the PnUGT94s from an individual *P. notoginseng* plant toward Rh1 with UDP-glucose as sugar donor.

(C) TLC analysis of the activities of the PnUGT94s from an individual *P. notoginseng* plant toward Rd with UDP-glucose as sugar donor.

(D) TLC analysis of the activities of the PnUGT94s from an individual *P. notoginseng* plant toward CK with UDP-glucose as sugar donor.

(E) TLC analysis of the activities of the PnUGT94s from an individual *P. notoginseng* plant toward F1 with UDP-glucose as sugar donor.

A

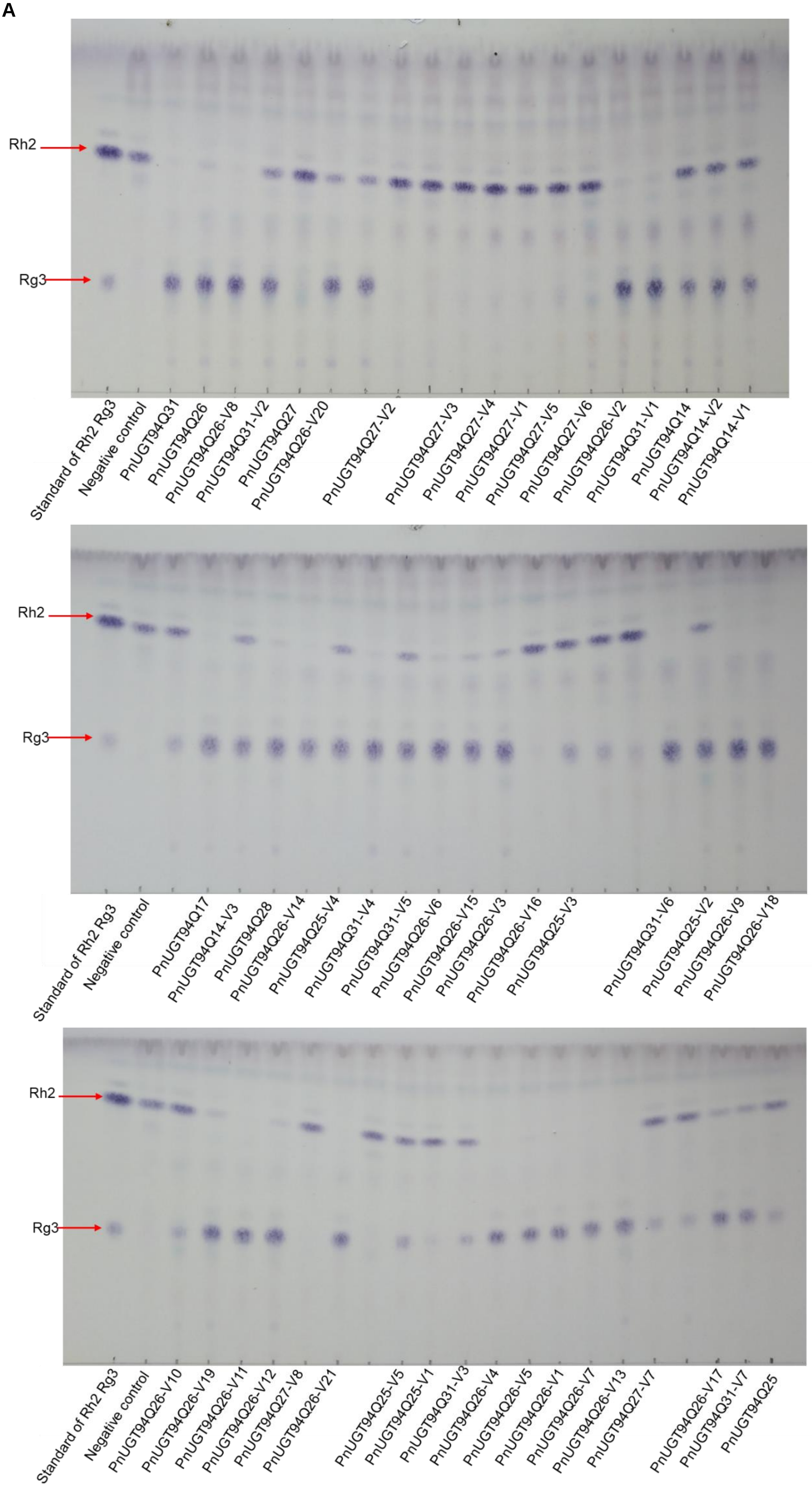
Rh2
Rg3
Standard of Rh2 Rg3
Negative control
PnUGT94Q31
PnUGT94Q26
PnUGT94Q26-V8
PnUGT94Q31-V2
PnUGT94Q27
PnUGT94Q26-V20
PnUGT94Q27-V2
PnUGT94Q27-V3
PnUGT94Q27-V4
PnUGT94Q27-V1
PnUGT94Q27-V5
PnUGT94Q27-V6
PnUGT94Q26-V2
PnUGT94Q31-V1
PnUGT94Q14
PnUGT94Q14-V2
PnUGT94Q14-V1
Rh2
Rg3
Standard of Rh2 Rg3
Negative control
PnUGT94Q17
PnUGT94Q14-V3
PnUGT94Q28
PnUGT94Q26-V14
PnUGT94Q25-V4
PnUGT94Q31-V4
PnUGT94Q31-V5
PnUGT94Q26-V6
PnUGT94Q26-V15
PnUGT94Q26-V3
PnUGT94Q26-V16
PnUGT94Q25-V3
PnUGT94Q31-V6
PnUGT94Q25-V2
PnUGT94Q26-V9
PnUGT94Q26-V18
Rh2
Rg3
Standard of Rh2 Rg3
Negative control
PnUGT94Q26-V10
PnUGT94Q26-V19
PnUGT94Q26-V11
PnUGT94Q26-V12
PnUGT94Q27-V8
PnUGT94Q26-V21
PnUGT94Q25-V5
PnUGT94Q25-V1
PnUGT94Q31-V3
PnUGT94Q26-V4
PnUGT94Q26-V5
PnUGT94Q26-V1
PnUGT94Q26-V7
PnUGT94Q26-V13
PnUGT94Q27-V7
PnUGT94Q26-V17
PnUGT94Q31-V7
PnUGT94Q25

B

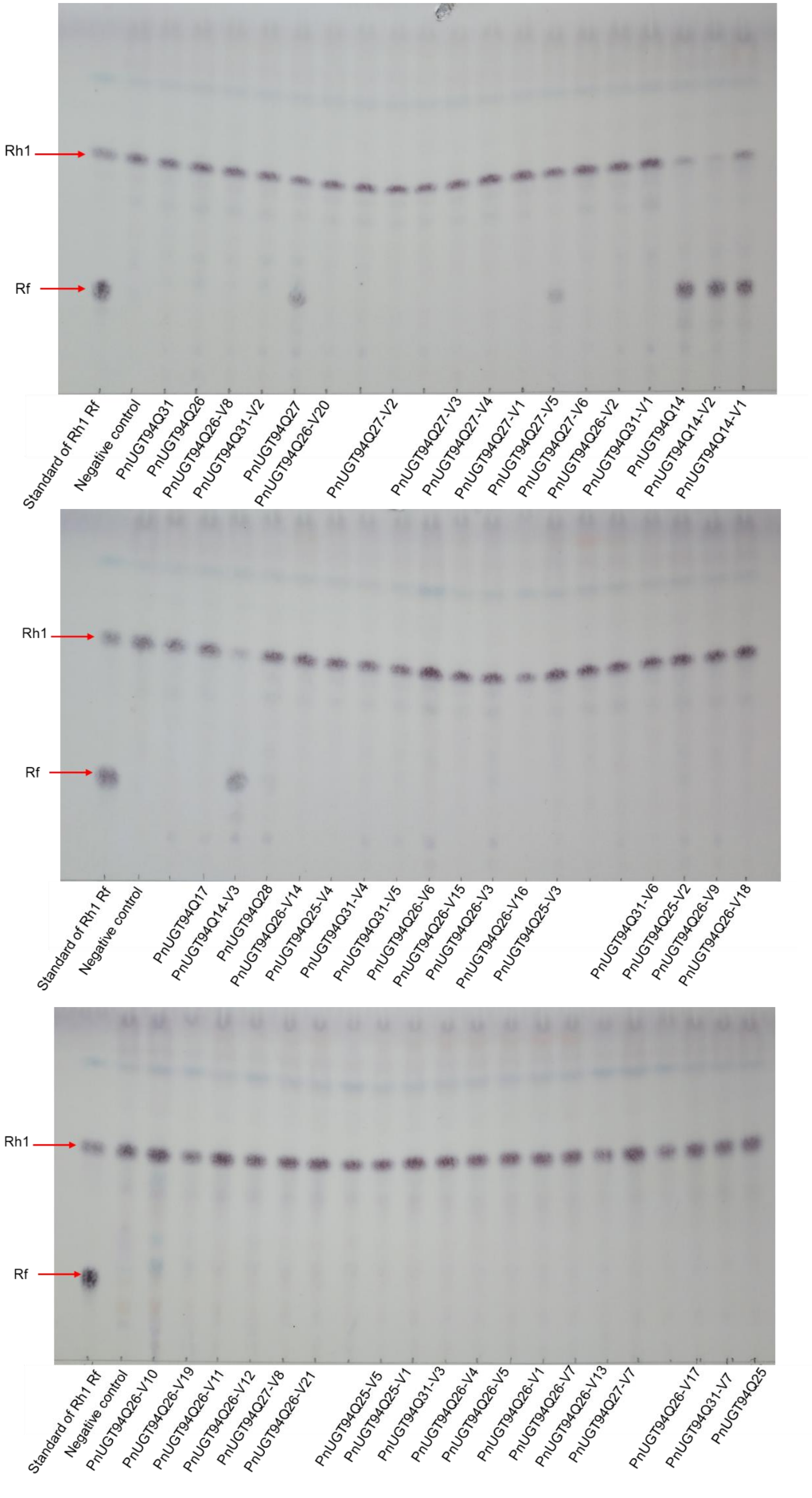
Rh1
Rf
Standard of Rh1 Rf
Negative control
PnUGT94Q31
PnUGT94Q26
PnUGT94Q26-V8
PnUGT94Q31-V2
PnUGT94Q27
PnUGT94Q26-V20
PnUGT94Q27-V2
PnUGT94Q27-V3
PnUGT94Q27-V4
PnUGT94Q27-V1
PnUGT94Q27-V5
PnUGT94Q27-V6
PnUGT94Q26-V2
PnUGT94Q31-V1
PnUGT94Q14
PnUGT94Q14-V2
PnUGT94Q14-V1
Rh1
Rf
Standard of Rh1 Rf
Negative control
PnUGT94Q17
PnUGT94Q14-V3
PnUGT94Q28
PnUGT94Q26-V14
PnUGT94Q25-V4
PnUGT94Q31-V4
PnUGT94Q31-V5
PnUGT94Q26-V6
PnUGT94Q26-V15
PnUGT94Q26-V3
PnUGT94Q26-V16
PnUGT94Q25-V3
PnUGT94Q31-V6
PnUGT94Q25-V2
PnUGT94Q26-V9
PnUGT94Q26-V18
Rh1
Rf
Standard of Rh1 Rf
Negative control
PnUGT94Q26-V10
PnUGT94Q26-V19
PnUGT94Q26-V11
PnUGT94Q26-V12
PnUGT94Q27-V8
PnUGT94Q26-V21
PnUGT94Q25-V5
PnUGT94Q25-V1
PnUGT94Q31-V3
PnUGT94Q26-V4
PnUGT94Q26-V5
PnUGT94Q26-V1
PnUGT94Q26-V7
PnUGT94Q26-V13
PnUGT94Q27-V7
PnUGT94Q26-V17
PnUGT94Q31-V7
PnUGT94Q25

C

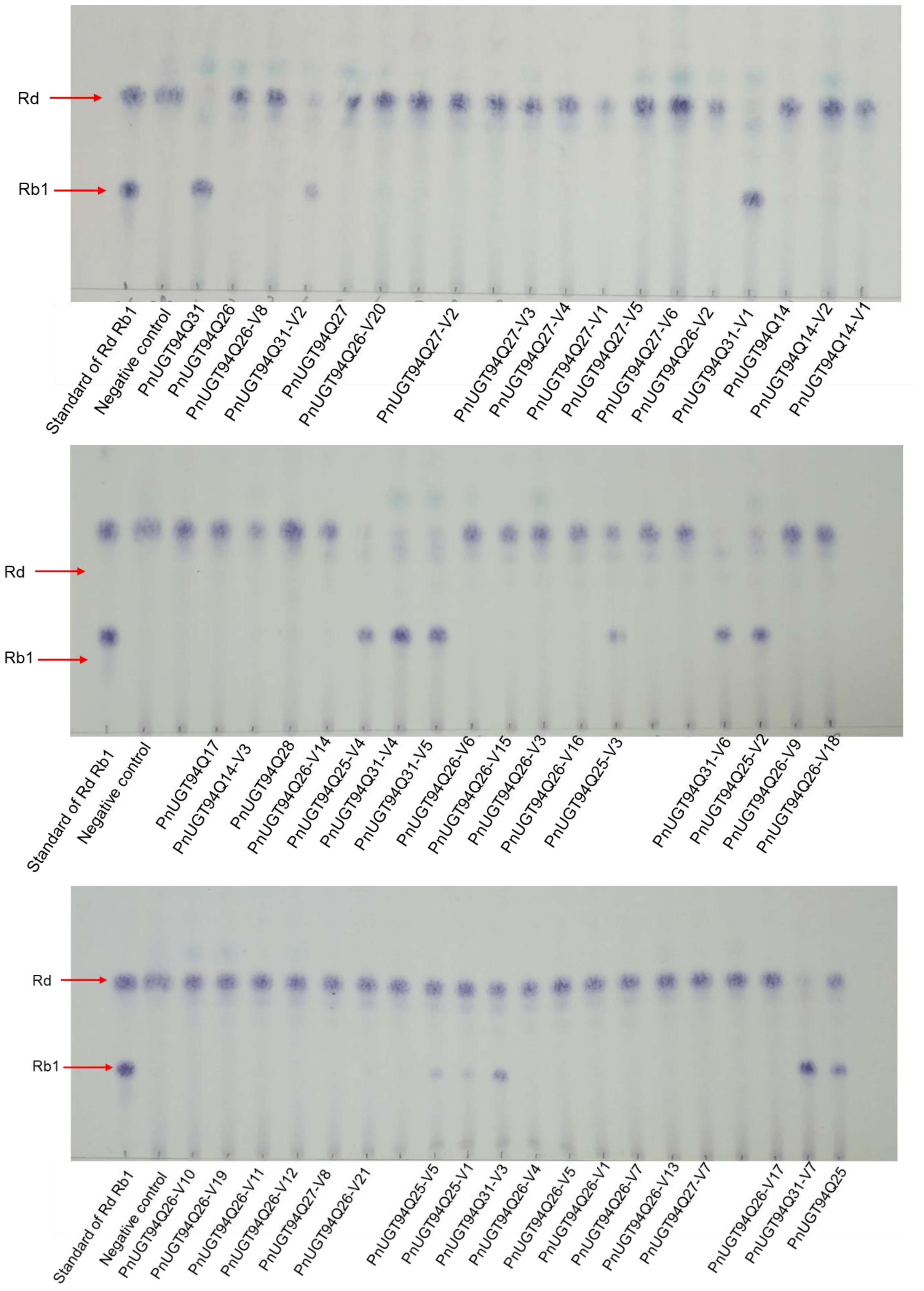
Rd
Rb1
Standard of Rd Rb1
Negative control
PnUGT94Q31
PnUGT94Q26
PnUGT94Q26-V8
PnUGT94Q31-V2
PnUGT94Q27
PnUGT94Q26-V20
PnUGT94Q27-V2
PnUGT94Q27-V3
PnUGT94Q27-V4
PnUGT94Q27-V1
PnUGT94Q27-V5
PnUGT94Q27-V6
PnUGT94Q26-V2
PnUGT94Q31-V1
PnUGT94Q14
PnUGT94Q14-V2
PnUGT94Q14-V1
Rd
Rb1
Standard of Rd Rb1
Negative control
PnUGT94Q17
PnUGT94Q14-V3
PnUGT94Q28
PnUGT94Q26-V14
PnUGT94Q25-V4
PnUGT94Q31-V4
PnUGT94Q31-V5
PnUGT94Q26-V6
PnUGT94Q26-V15
PnUGT94Q26-V3
PnUGT94Q26-V16
PnUGT94Q25-V3
PnUGT94Q31-V6
PnUGT94Q25-V2
PnUGT94Q26-V9
PnUGT94Q26-V18
Rd
Rb1
Standard of Rd Rb1
Negative control
PnUGT94Q26-V10
PnUGT94Q26-V19
PnUGT94Q26-V11
PnUGT94Q26-V12
PnUGT94Q27-V8
PnUGT94Q26-V21
PnUGT94Q25-V5
PnUGT94Q25-V1
PnUGT94Q31-V3
PnUGT94Q26-V4
PnUGT94Q26-V5
PnUGT94Q26-V1
PnUGT94Q26-V7
PnUGT94Q26-V13
PnUGT94Q27-V7
PnUGT94Q26-V17
PnUGT94Q31-V7
PnUGT94Q25

D

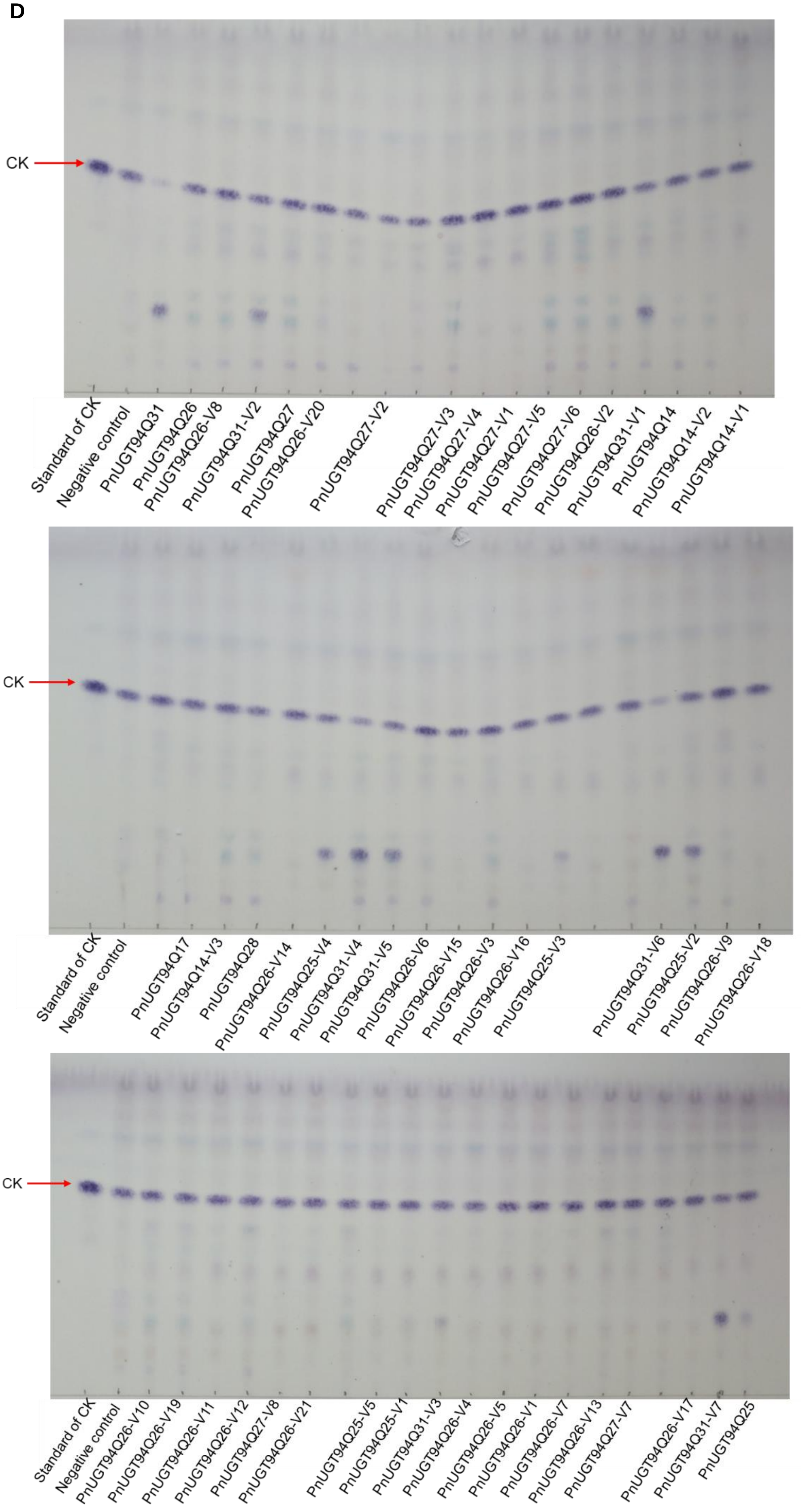
CK
Standard of CK
Negative control
PnUGT94Q31
PnUGT94Q26
PnUGT94Q26-V8
PnUGT94Q31-V2
PnUGT94Q27
PnUGT94Q26-V20
PnUGT94Q27-V2
PnUGT94Q27-V3
PnUGT94Q27-V4
PnUGT94Q27-V1
PnUGT94Q27-V5
PnUGT94Q27-V6
PnUGT94Q26-V2
PnUGT94Q31-V1
PnUGT94Q14
PnUGT94Q14-V2
PnUGT94Q14-V1
CK
Standard of CK
Negative control
PnUGT94Q17
PnUGT94Q14-V3
PnUGT94Q28
PnUGT94Q26-V14
PnUGT94Q25-V4
PnUGT94Q31-V4
PnUGT94Q31-V5
PnUGT94Q26-V6
PnUGT94Q26-V15
PnUGT94Q26-V3
PnUGT94Q26-V16
PnUGT94Q25-V3
PnUGT94Q31-V6
PnUGT94Q25-V2
PnUGT94Q26-V9
PnUGT94Q26-V18
CK
Standard of CK
Negative control
PnUGT94Q26-V10
PnUGT94Q26-V19
PnUGT94Q26-V11
PnUGT94Q26-V12
PnUGT94Q27-V8
PnUGT94Q26-V21
PnUGT94Q25-V5
PnUGT94Q25-V1
PnUGT94Q31-V3
PnUGT94Q26-V4
PnUGT94Q26-V5
PnUGT94Q26-V1
PnUGT94Q26-V7
PnUGT94Q26-V13
PnUGT94Q27-V7
PnUGT94Q26-V17
PnUGT94Q31-V7
PnUGT94Q25

E

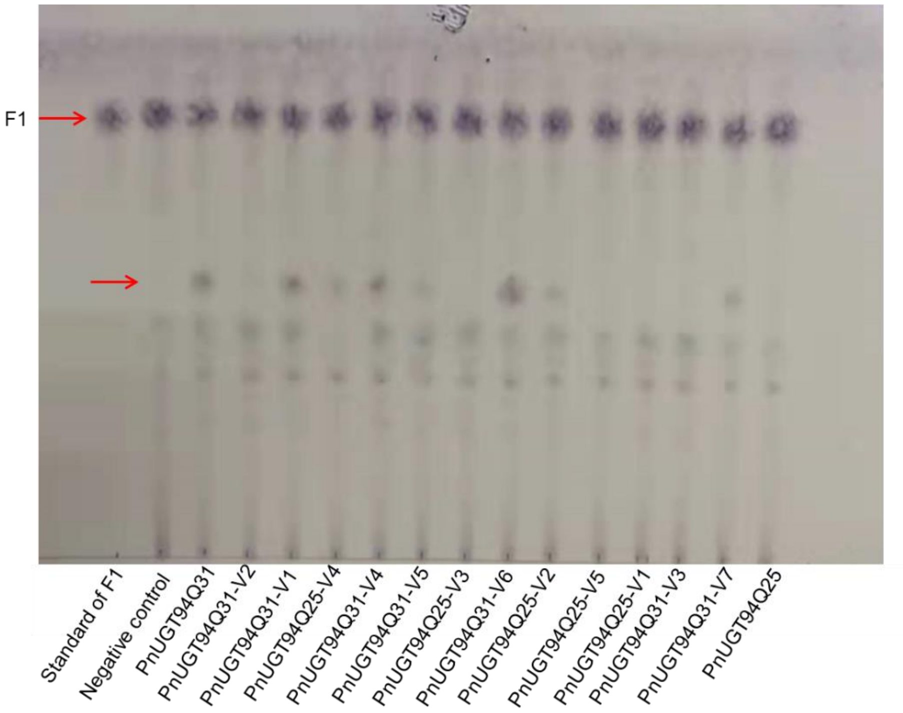

F1
Standard of F1
Negative control
PnUGT94Q31
PnUGT94Q31-V2
PnUGT94Q31-V1
PnUGT94Q25-V4
PnUGT94Q31-V4
PnUGT94Q31-V5
PnUGT94Q25-V3
PnUGT94Q31-V6
PnUGT94Q25-V2
PnUGT94Q25-V5
PnUGT94Q25-V1
PnUGT94Q31-V3
PnUGT94Q31-V7
PnUGT94Q25

Figure S4. TLC analysis of the activities of the PnUGT94s toward Rh2, Rh1,and Rd with UDP-glucose as sugar donor.

(A) TLC analysis of the activities of the PnUGT94s from other individual *P. notoginseng* plant toward Rh2 with UDP-glucose as sugar donor.

(B) TLC analysis of the activities of the PnUGT94s from other individual *P. notoginseng* plant toward Rh1 with UDP-glucose as sugar donor.

(C) TLC analysis of the activities of the PnUGT94s from other individual *P. notoginseng* plant toward Rd with UDP-glucose as sugar donor.

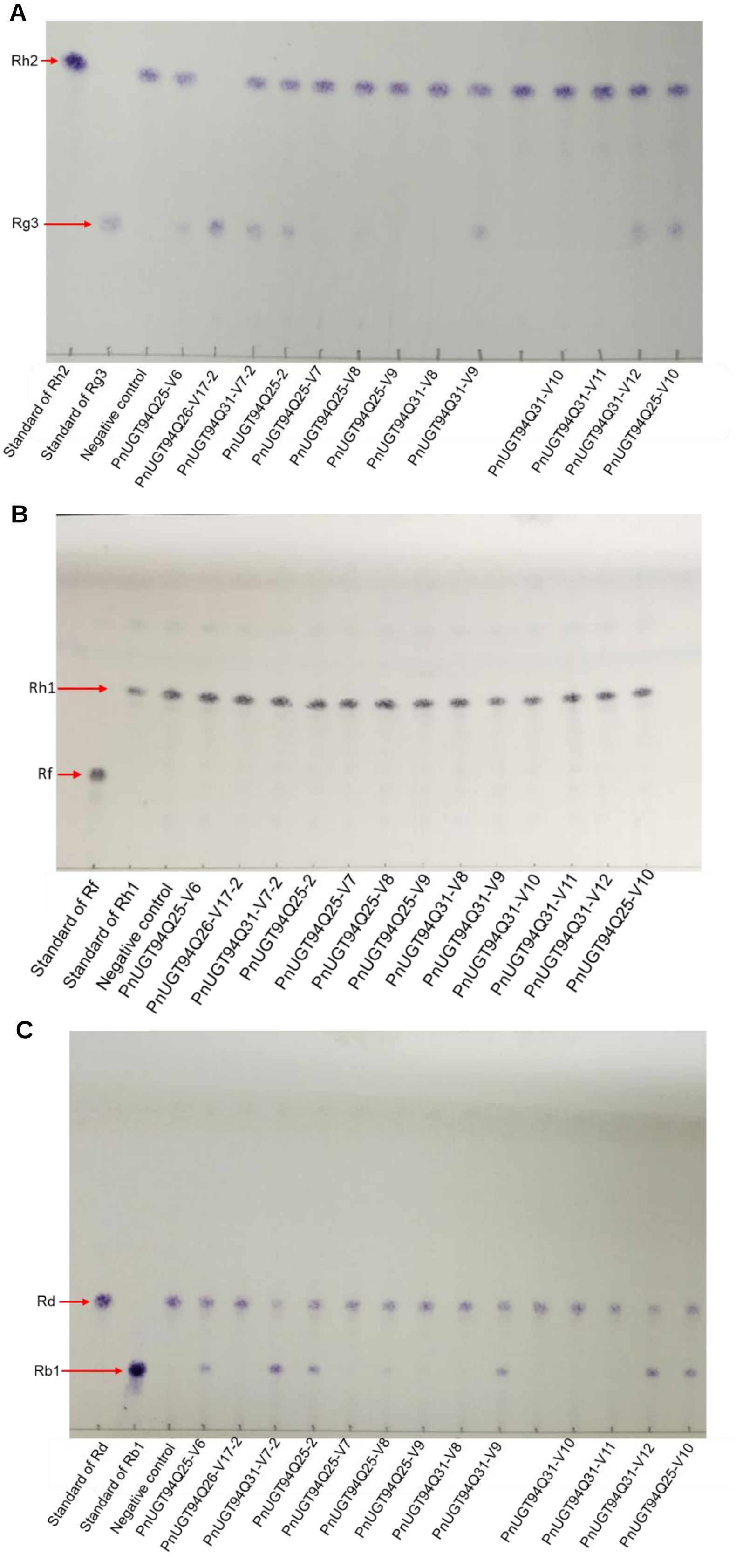

Figure S5. The sequences alignment of cloned and assembled scaffolds which contain UGT94 homologue genes in the *P. ginseng* genome

(A) The sequences alignment of cloned sequences of 14.5 to 16.0 kb of Pg_scaffold6708, 14.5 to 16.0 kb of Pg_scaffold6708 by Nam-Hoon Kim et.al, 2018, and 14.7 to 16.2 kb of Scaffold8624 by Jiang Xu et.al, 2017.

(B) The sequences alignment of cloned sequences of 24.1 to 25.8 kb of Pg_scaffold6708, 24.1 to 25.8 kb of Pg_scaffold6708 by Nam-Hoon Kim et.al, 2018, and 25.6 to 27.4 kb of Scaffold8624 by Jiang Xu et.al, 2017.

(C) The sequences alignment of cloned sequences of 19.6 to 21.3 kb of Pg_scaffold6708, 19.6 to 21.3 kb of Pg_scaffold6708 by Nam-Hoon Kim et.al, 2018, and 19.8 to 22.8 kb of Scaffold8624 by Jiang Xu et.al, 2017.

(D) The sequences alignment of cloned sequences of 418.1 to 419.5 kb of Pg_scaffold2289, 13.1 to 14.9 kb of Scaffold9344 by Jiang Xu et.al, 2017, and 418.1 to 419.5 kb of Pg_scaffold2289 by Nam-Hoon Kim et.al, 2018.

(E) The sequences alignment of cloned sequences of 430.4 to 431.9 kb of Pg_scaffold2289, 15.2 to 16.7 kb of Scaffold2205 by Jiang Xu et.al, 2017, and 430.4 to 431.9 kb of Pg_scaffold2289 by Nam-Hoon Kim et.al, 2018.

A

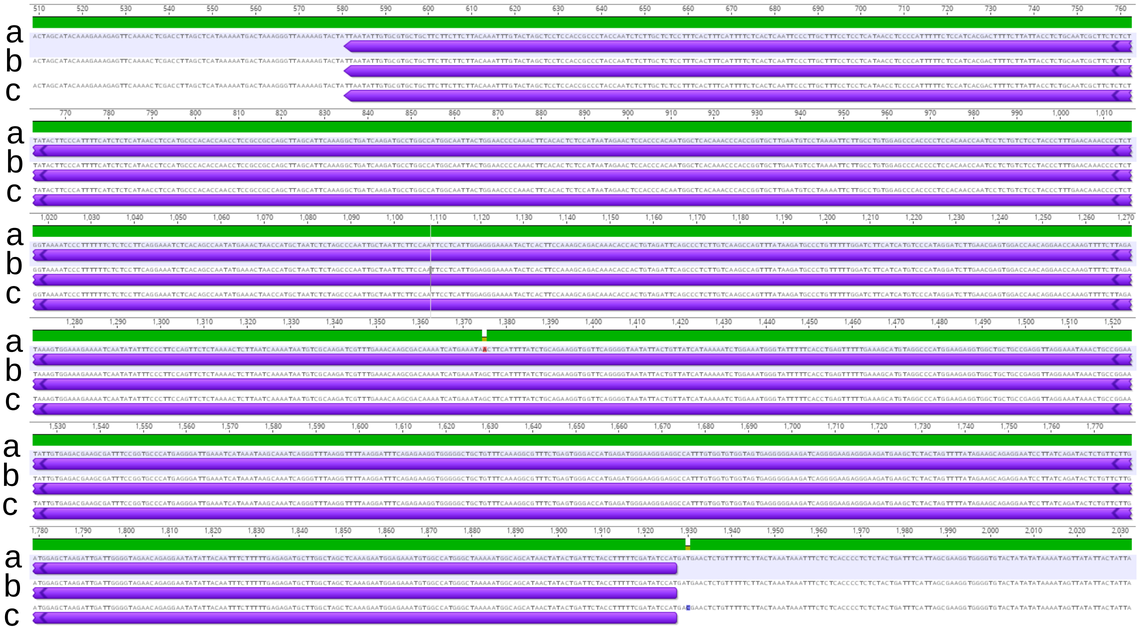

a: The cloned sequences of 14.5 to 16.0 kb of Pg_scaffold6708.
b: The assembled sequences of 14.5 to 16.0 kb of Pg_scaffold6708 by Nam-Hoon Kim et.al, 2018.
c: The assembled sequences of 14.7 to 16.2 kb of Scaffold8624 by Jiang Xu et.al, 2017.

B

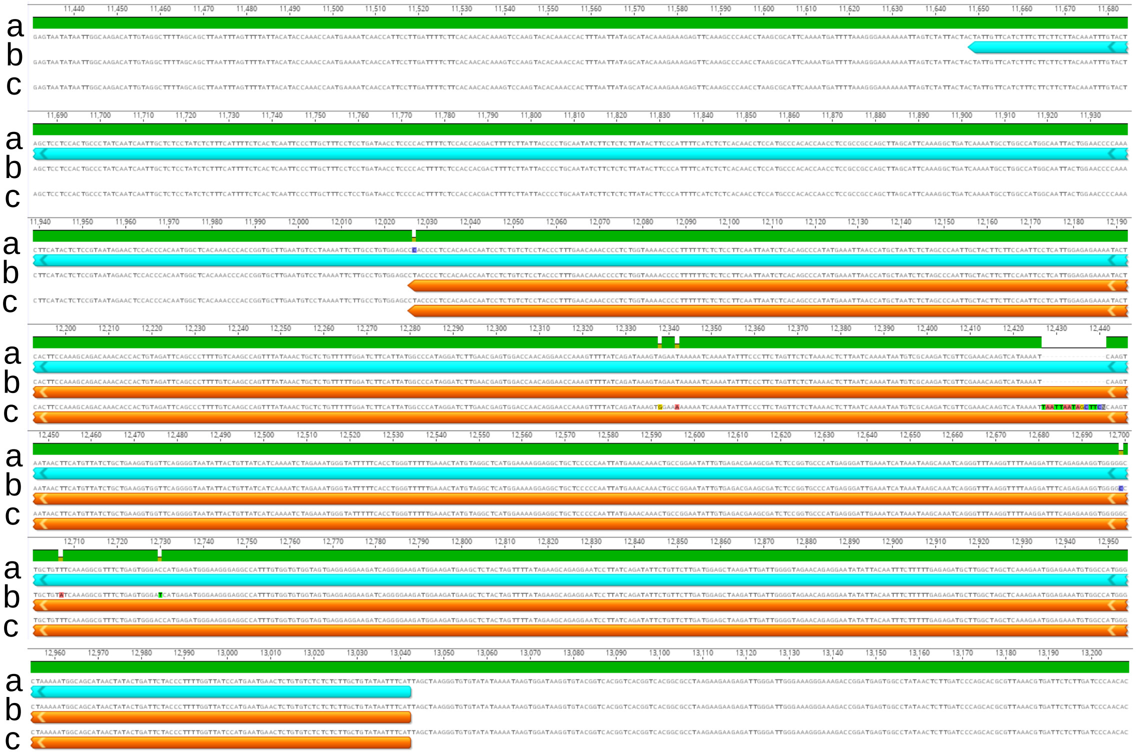

a: The cloned sequences of 24.1 to 25.8 kb of Pg_scaffold6708.
b: The assembled sequences of 24.1 to 25.8 kb of Pg_scaffold6708 by Nam-Hoon Kim et.al, 2018.
c: The assembled sequences of 25.6 to 27.4 kb of Scaffold8624 by Jiang Xu et.al, 2017.

c

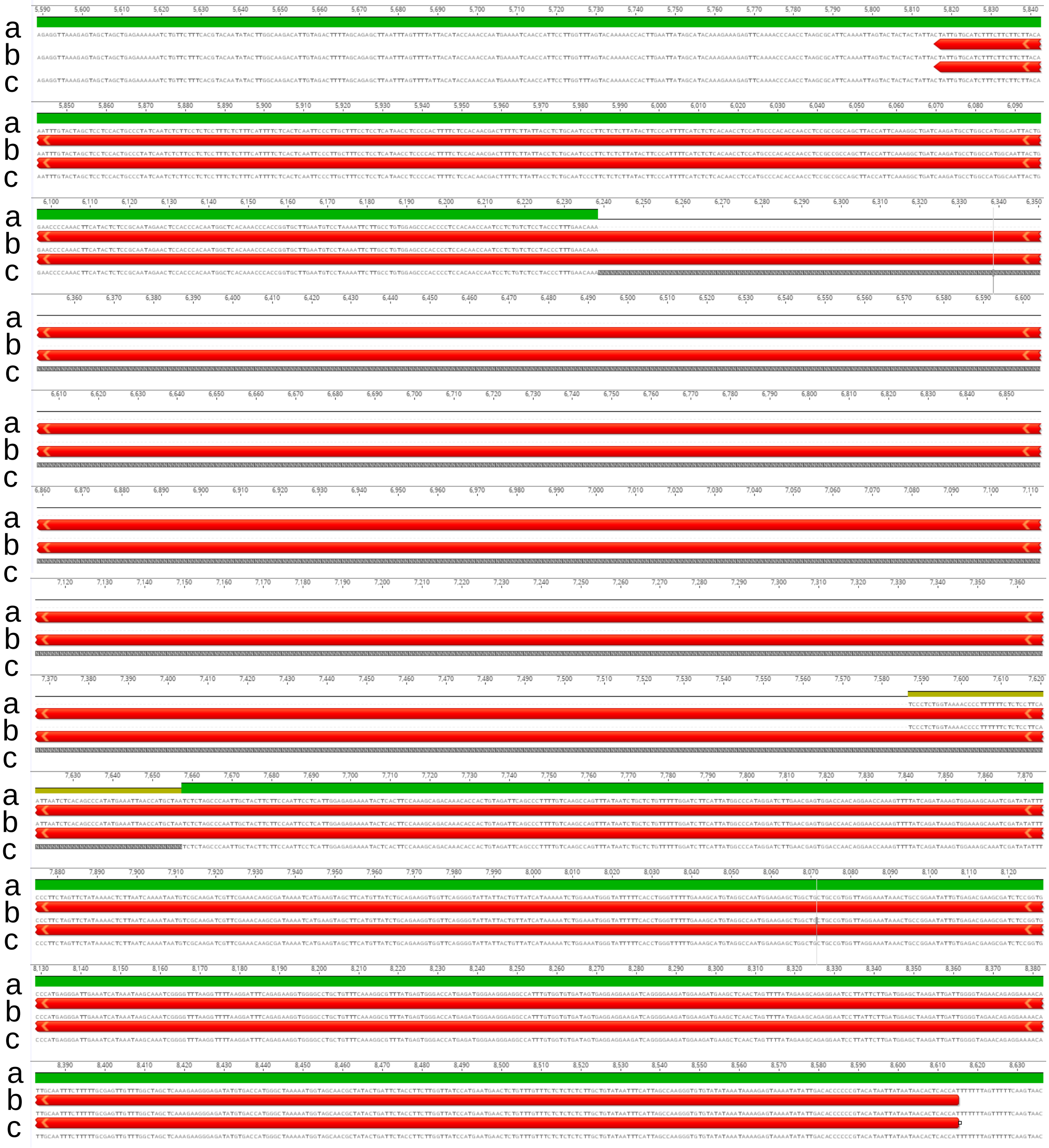

a: The cloned sequences of 19.6 to 21.3 kb of Pg_scaffold6708.

b: The assembled sequences of 19.6 to 21.3 kb of Pg_scaffold6708 by Nam-Hoon Kim et.al, 2018.

c: The assembled sequences of 19.8 to 22.8 kb of Scaffold8624 by Jiang Xu et.al, 2017.

D

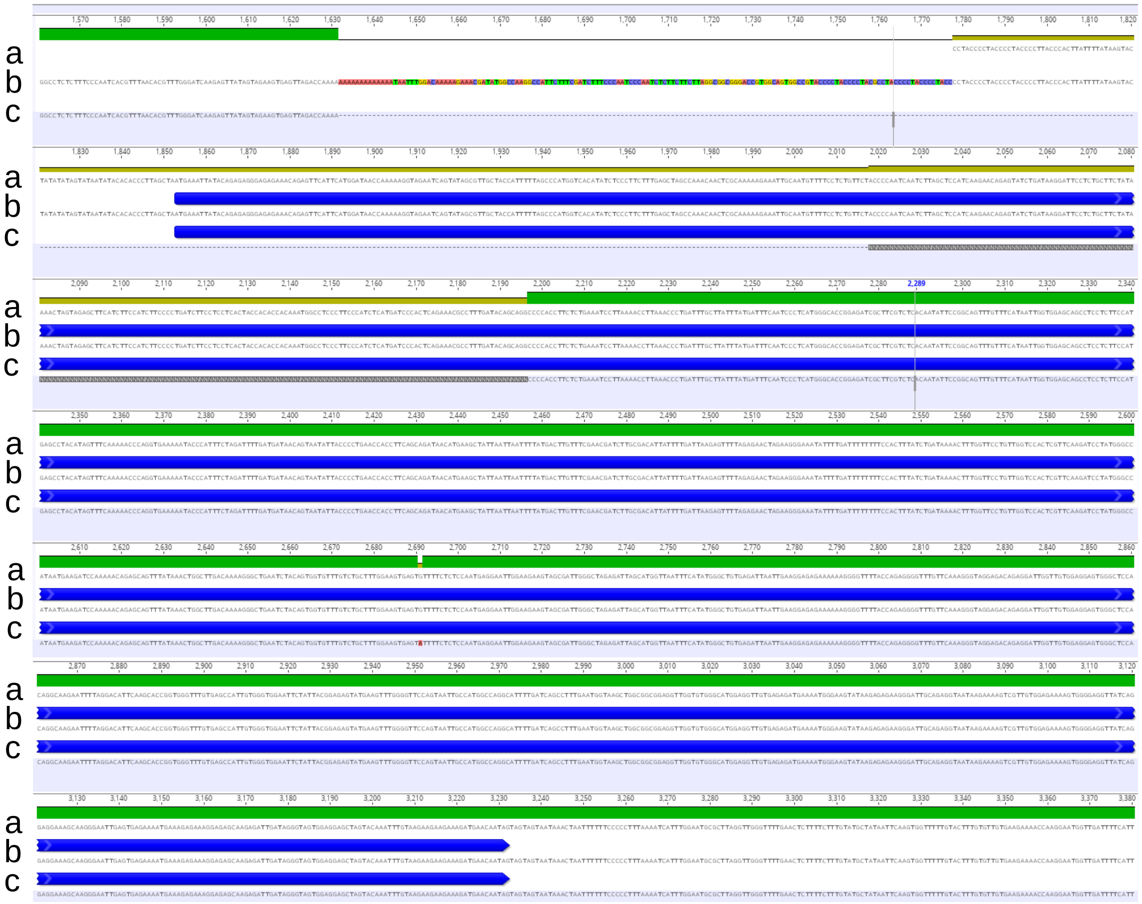

a: The cloned sequences of 418.1 to 419.5 kb of Pg_scaffold2289.
b: The assembled sequences of 13.1 to 14.9 kb of Scaffold9344 by Jiang Xu et.al, 2017.
c: The assembled sequences of 418.1 to 419.5 kb of Pg_scaffold2289 by Nam-Hoon Kim et.al, 2018.

E

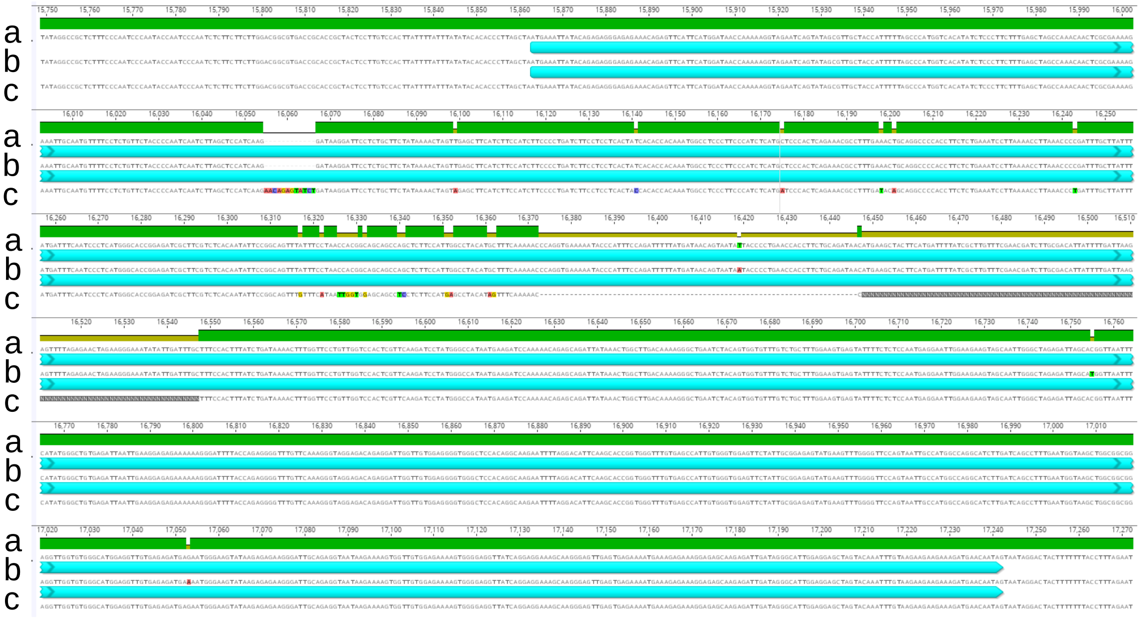

a: The cloned sequences of 430.4 to 431.9 kb of Pg_scaffold2289.
b: The assembled sequences of 15.2 to 16.7 kb of Scaffold2205 by Jiang Xu et.al, 2017.
c: The assembled sequences of 430.4 to 431.9 kb of Pg_scaffold2289 by Nam-Hoon Kim et.al, 2018.

Figure S6. Phylogenetic analysis of deduced amino acid sequences of the UGT94s from *P. ginseng* and *P. notoginseng*. Only bootstrap values above 70% are shown in the tree.

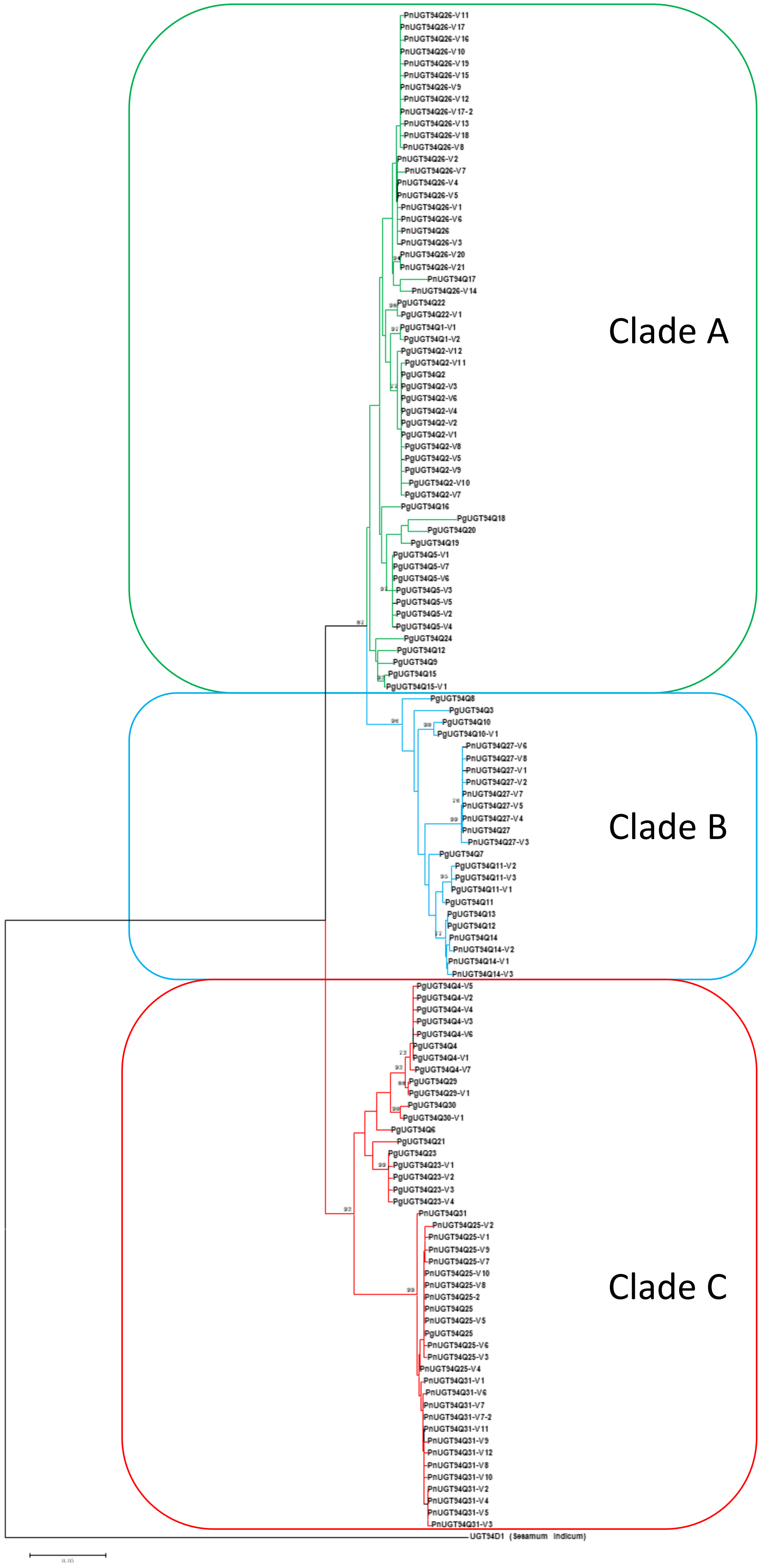

Figure S7. HR-ESIMS analysis of Rg3, Rf, Rb1, Rd, gypenoside LXXV, Zingibroside R1, 3-*O*-[*β*-D-glucopyranosyl-(1→2)-*β*-D-glucopyranosyl]-oleanolic acid, and notoginsenoside U.

(A) The MS spectrum of Rg3 produced by an *in vitro* reaction catalyzed by PgUGT94Q15 toward Rh2 with UDP-glucose as sugar donor.

(B) The MS spectrum of standard of Rg3.

(C) The MS spectrum of Rf produced by an *in vitro* reaction catalyzed by PgUGT94Q3 toward Rh1 with UDP-glucose as sugar donor.

(D) The MS spectrum of standard of Rf.

(E) The MS spectrum of Rb1 produced by an *in vitro* reaction catalyzed by PgUGT94Q6 toward Rd with UDP-glucose as sugar donor.

(F) The MS spectrum of standard of Rb1.

(G) The MS spectrum of Rd produced by an *in vitro* reaction catalyzed by PgUGT94Q15 toward F2 with UDP-glucose as sugar donor.

(H) The MS spectrum of standard of Rd.

(I) The MS spectrum of Gypenoside LXXV produced by an *in vitro* reaction catalyzed by PgUGT94Q6 toward CK with UDP-glucose as sugar donor.

(J) The MS spectrum of standard of Zingibroside R1.

(K) The MS spectrum of Zingibroside R1 produced by an *in vitro* reaction catalyzed by PgUGT94Q15-V1 toward Calenduloside E with UDP-glucose as sugar donor.

(L) The MS spectrum of 3-*O*-[*β*-D-glucopyranosyl-(1→2)-*β*-D-glucopyranosyl]-oleanolic acid produced by an *in vitro* reaction catalyzed by PgUGT94Q15 toward 3GOA with UDP-glucose as sugar donor.

(M) The MS spectrum of notoginsenoside U produced by an *in vitro* reaction catalyzed by PgUGT94Q6 toward F1 with UDP-glucose as sugar donor.

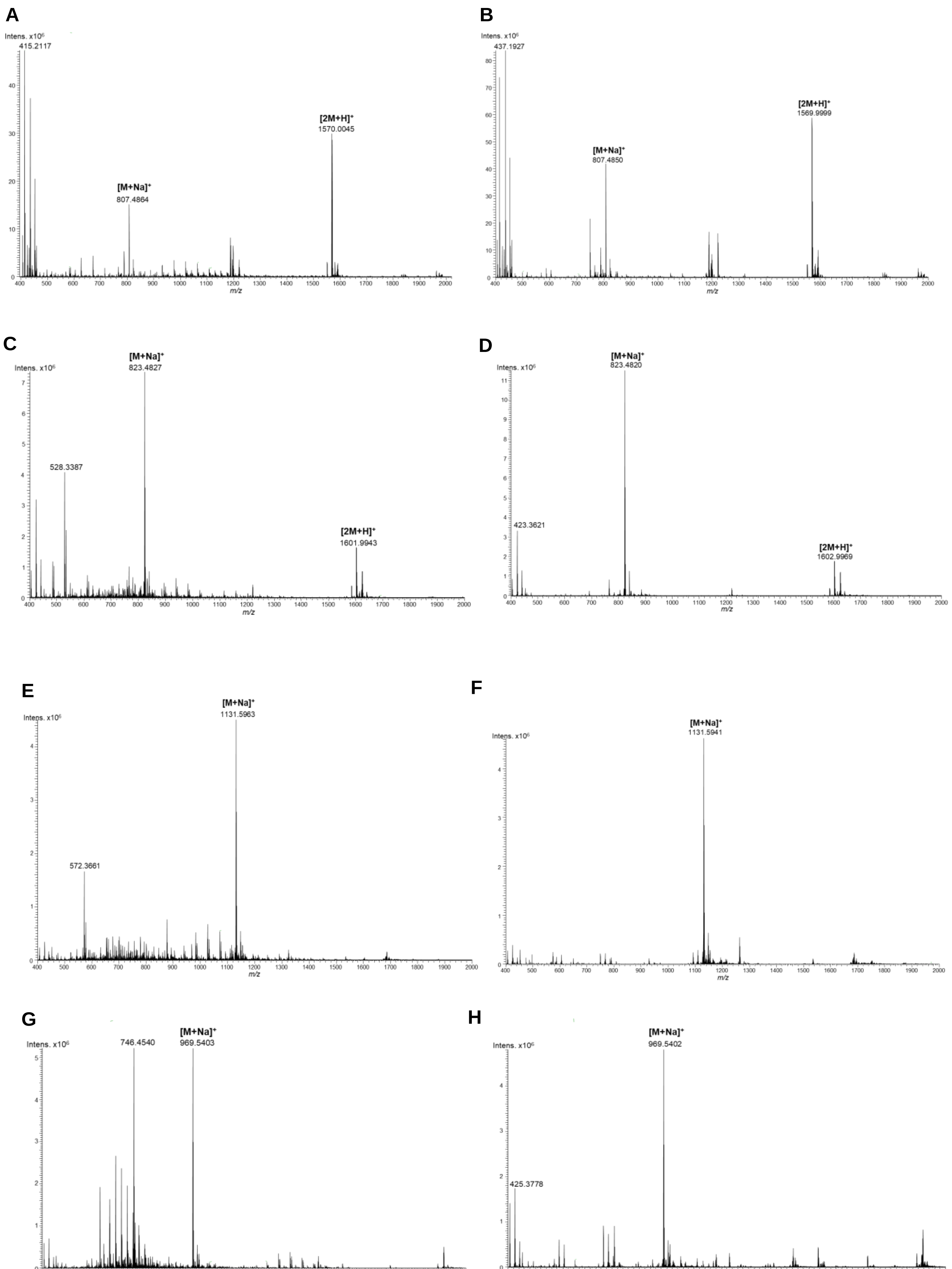

A
Intens. x10^6
415.2117
[M+Na]+
807.4864
[2M+H]+
1570.0045
m/z
B
Intens. x10^6
437.1927
[M+Na]+
807.4850
[2M+H]+
1569.9999
m/z
C
Intens. x10^6
[M+Na]+
823.4827
528.3387
[2M+H]+
1601.9943
m/z
D
Intens. x10^6
[M+Na]+
823.4820
423.3621
[2M+H]+
1602.9969
m/z
E
Intens. x10^6
[M+Na]+
1131.5963
572.3661
m/z
F
Intens. x10^6
[M+Na]+
1131.5941
m/z
G
Intens. x10^6
746.4540
[M+Na]+
969.5403
m/z
H
Intens. x10^6
[M+Na]+
969.5402
425.3778
m/z

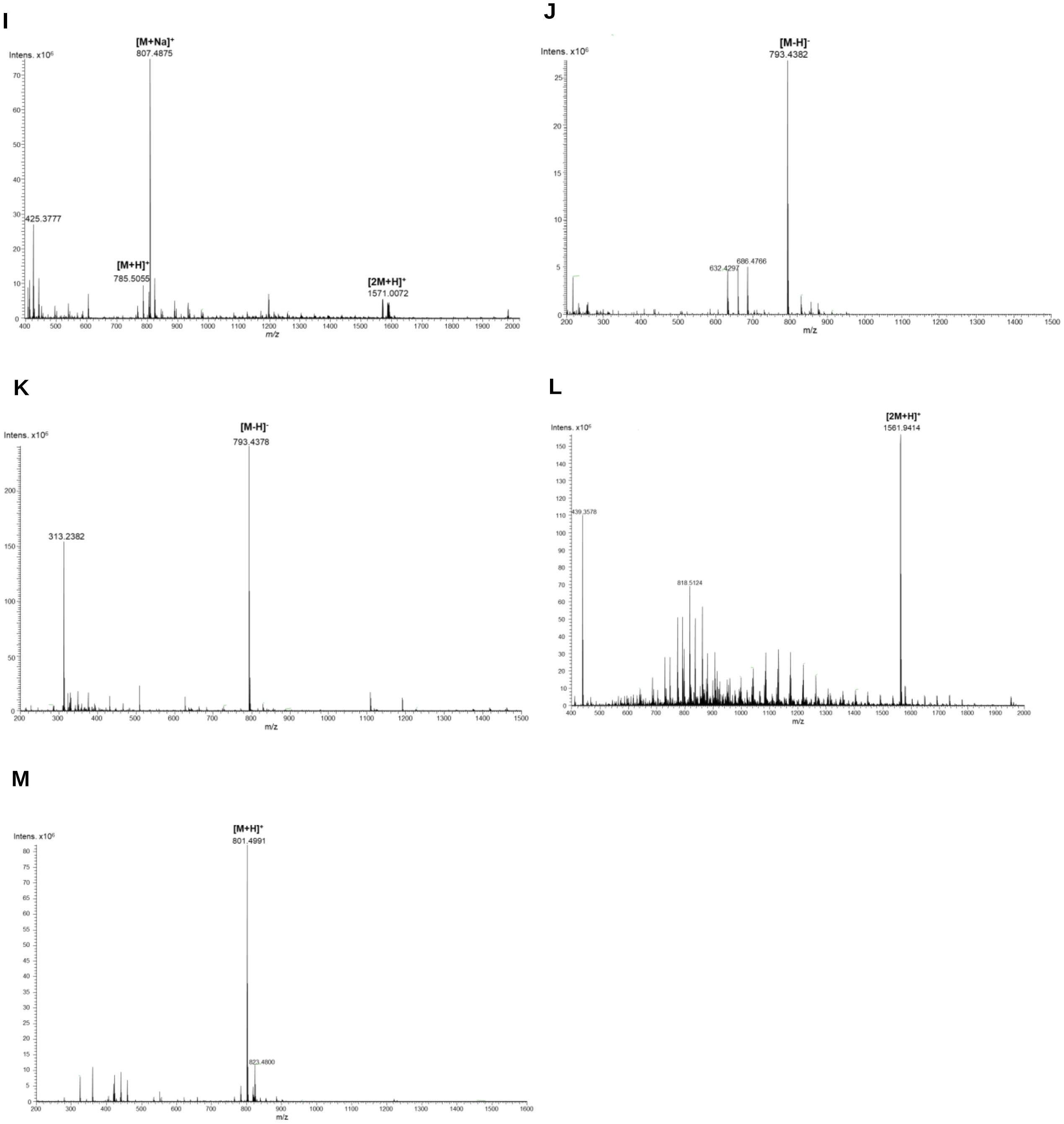

I
[M+Na]+
807.4875
Intens. x10^6
425.3777
[M+H]+
785.5055
[2M+H]+
1571.0072
m/z
J
[M-H]-
793.4382
Intens. x10^6
632.4297
686.4766
m/z
K
[M-H]-
793.4378
Intens. x10^6
313.2382
m/z
L
[2M+H]+
1561.9414
Intens. x10^6
439.3578
818.5124
m/z
M
[M+H]+
801.4991
Intens. x10^6
823.4800
m/z

Figure S8. NMR analysis of Rg3, Rf, Rb1, notoginsenoside U, gypenoside LXXV, and 3-*O*-[*β*-D-glucopyranosyl-(1→2)-*β*-D-glucopyranosyl]-oleanolic acid.

(A) ^{1}H-NMR spectrum of Rg3.
(B) ^{1}H-NMR spectrum of Rf.
(C) ^{13}C-NMR spectrum of Rf.
(D) ^{1}H-NMR spectrum of Rb1.
(E) ^{1}H-NMR spectrum of notoginsenoside U.
(F) ^{1}H-NMR spectrum of gypenoside LXXV.
(G) ^{13}C-NMR spectrum of gypenoside LXXV.
(H) HMBC spectrum of gypenoside LXXV.
(I) HSQC spectrum of gypenoside LXXV.
(J) ^{1}H-NMR spectrum of 3-*O*-[*β*-D-glucopyranosyl-(1→2)-*β*-D-glucopyranosyl]-oleanolic acid.
(K) ^{13}C-NMR spectrum of 3-*O*-[*β*-D-glucopyranosyl-(1→2)-*β*-D-glucopyranosyl]-oleanolic acid.
(L) HSQC spectrum of 3-*O*-[*β*-D-glucopyranosyl-(1→2)-*β*-D-glucopyranosyl]-oleanolic acid.
(M) ^{1}H-^{1}H correlation spectroscopy of 3-*O*-[*β*-D-glucopyranosyl-(1→2)-*β*-D-glucopyranosyl]-oleanolic acid.
(N) HMBC spectrum of 3-*O*-[*β*-D-glucopyranosyl-(1→2)-*β*-D-glucopyranosyl]-oleanolic acid.
(O) NOESY spectrum of 3-*O*-[*β*-D-glucopyranosyl-(1→2)-*β*-D-glucopyranosyl]-oleanolic acid.

A

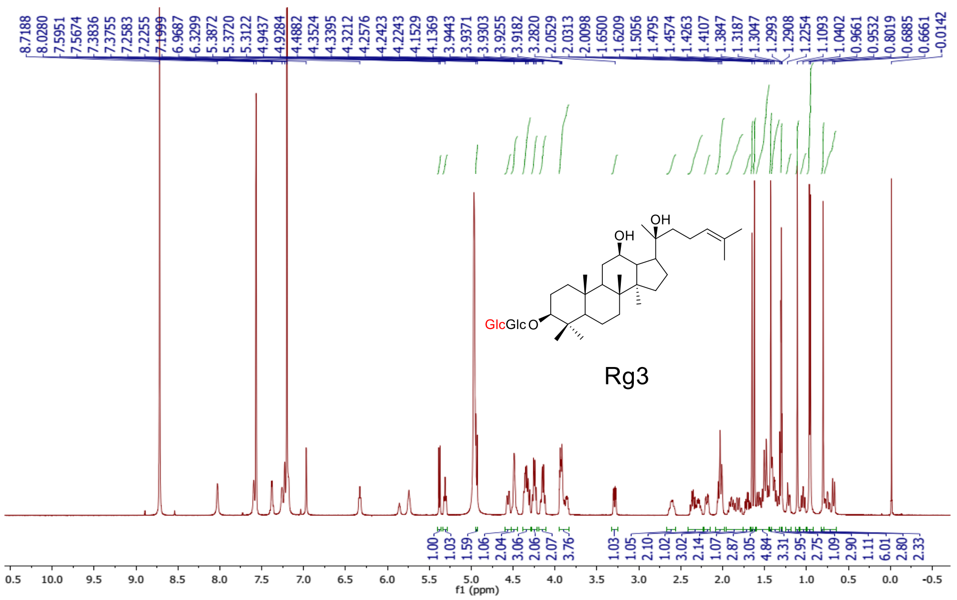

Rg3
GlcGlc O
OH
f1 (ppm)

B

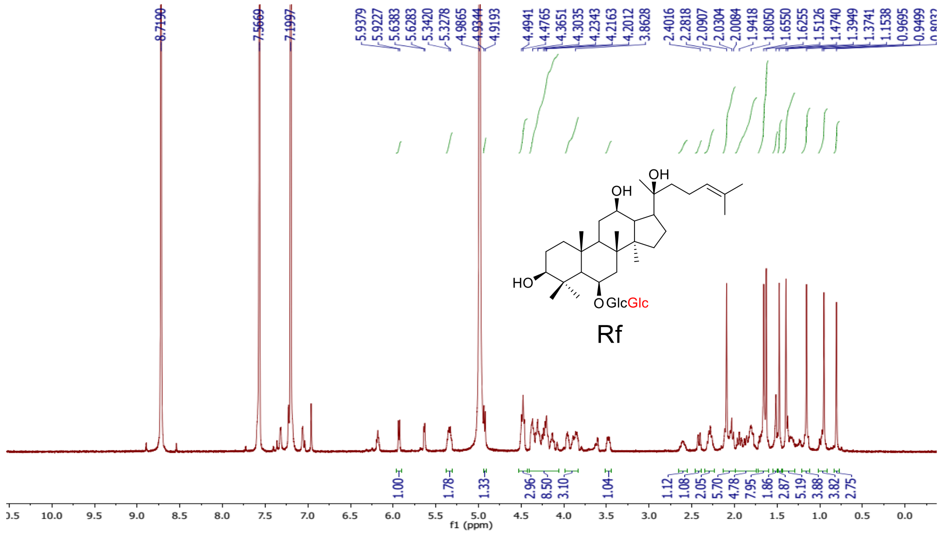

Rf
HO
OGlcGlc
OH
f1 (ppm)

C

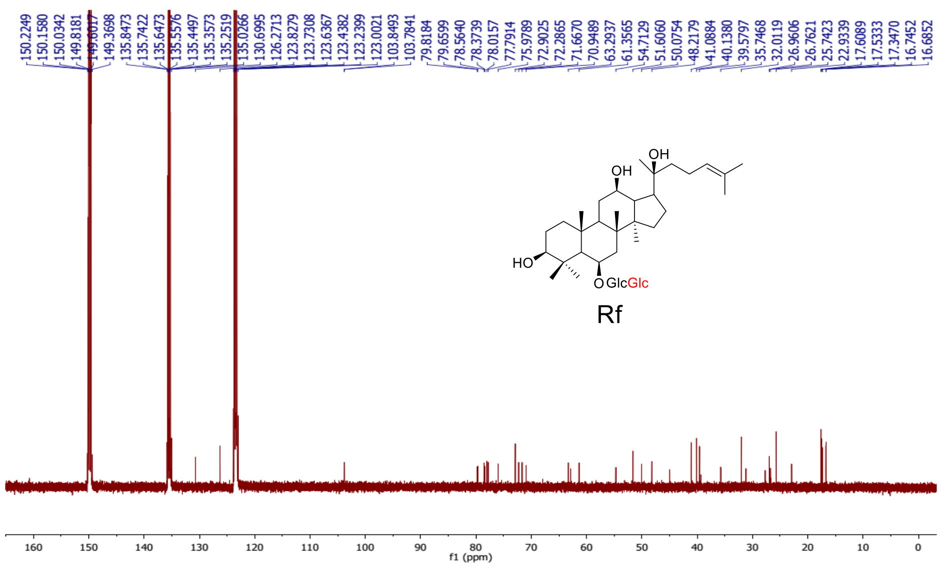

Rf
HO
OGlcGlc
OH
f1 (ppm)

D

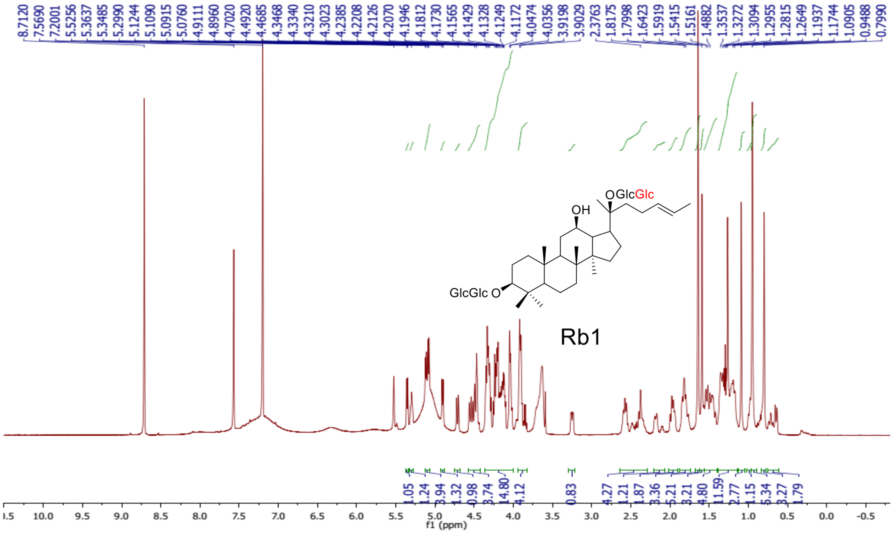

E

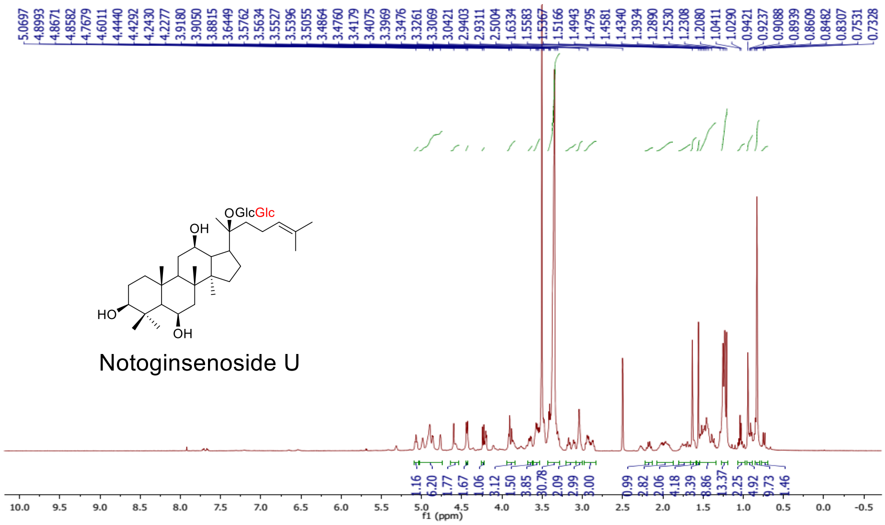

F

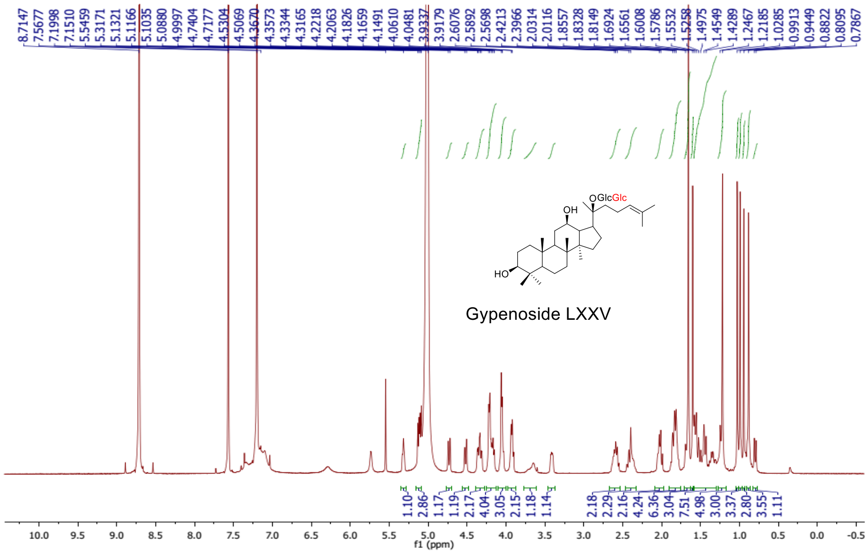

G

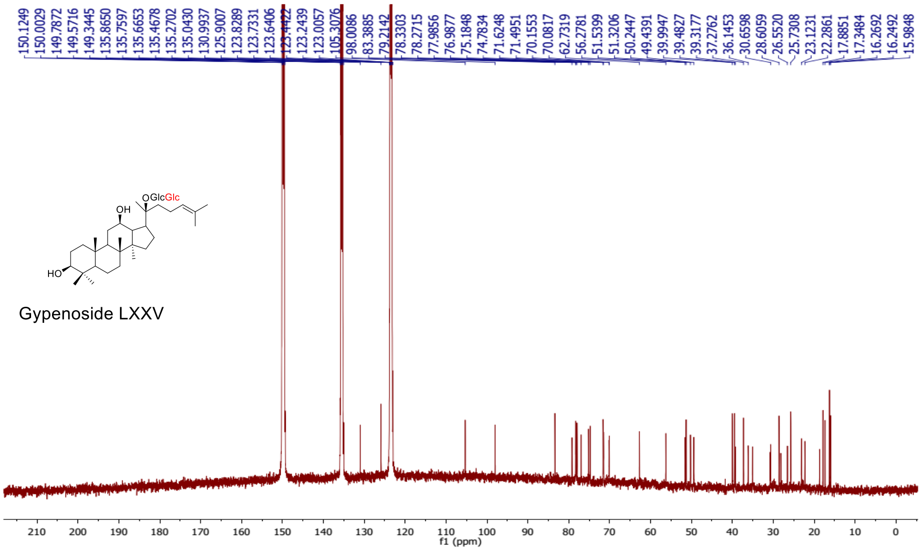
150.1249
150.0029
149.7872
149.5716
149.3445
135.8650
135.7597
135.6653
135.4678
135.2702
135.0430
130.9937
125.9007
123.8289
123.7331
123.6406
123.4422
123.2439
123.0057
105.3076
98.0086
83.3885
79.2142
78.3303
78.2715
77.9856
76.9877
75.1848
74.7834
71.6248
71.4951
70.1553
70.0817
62.7319
56.2781
51.5399
51.3206
50.2447
49.4391
39.9947
39.4827
39.3177
37.2762
36.1453
30.6598
28.6059
26.5520
25.7308
23.1231
22.2861
17.8851
17.3484
16.2692
16.2492
15.9848
OGlcGlc
OH
HO
Gypenoside LXXV
f1 (ppm)

H

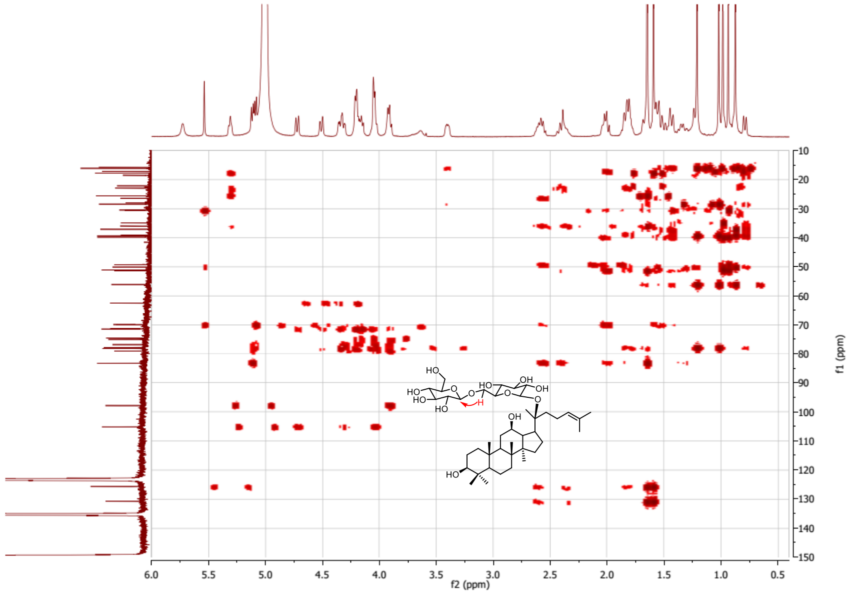
HO
OH
f2 (ppm)
f1 (ppm)

I

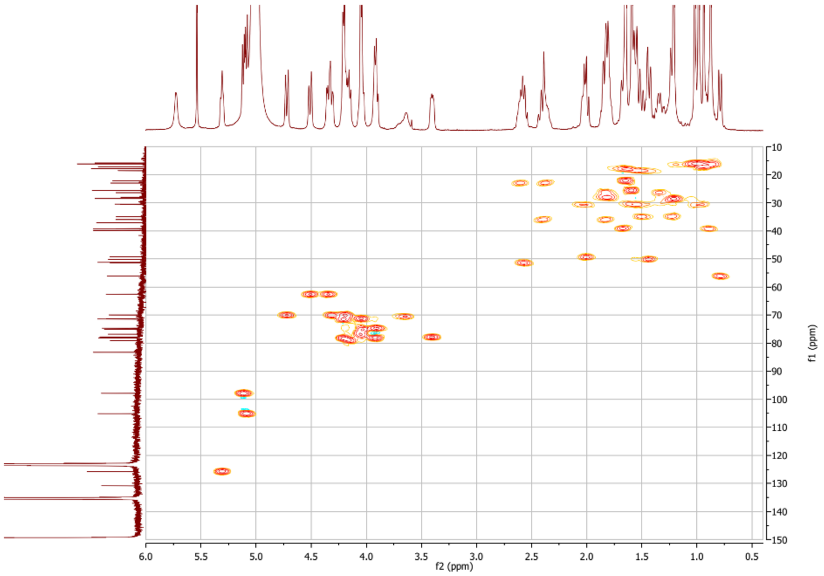
f2 (ppm)
f1 (ppm)

J

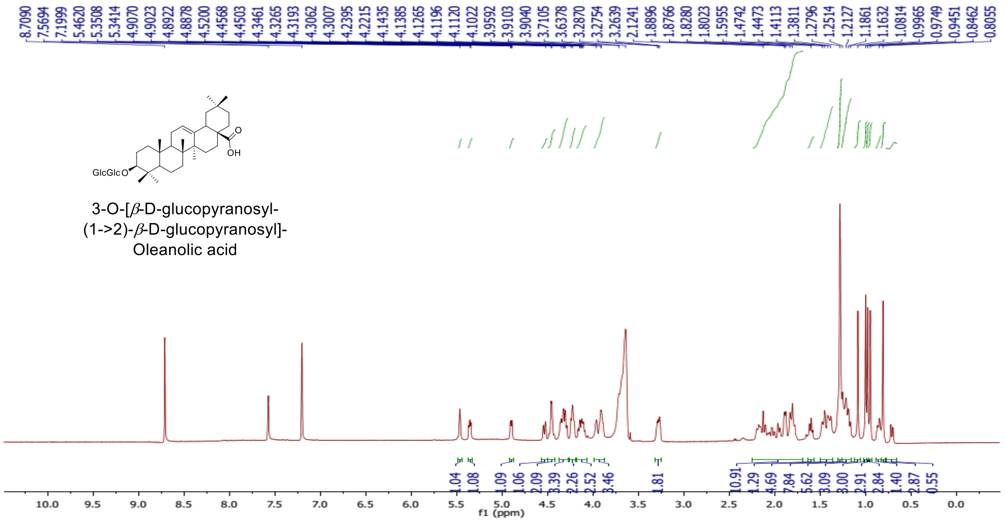
GlcGlcO
OH
3-O-[β-D-glucopyranosyl-(1->2)-β-D-glucopyranosyl]-Oleanolic acid
f1 (ppm)

K

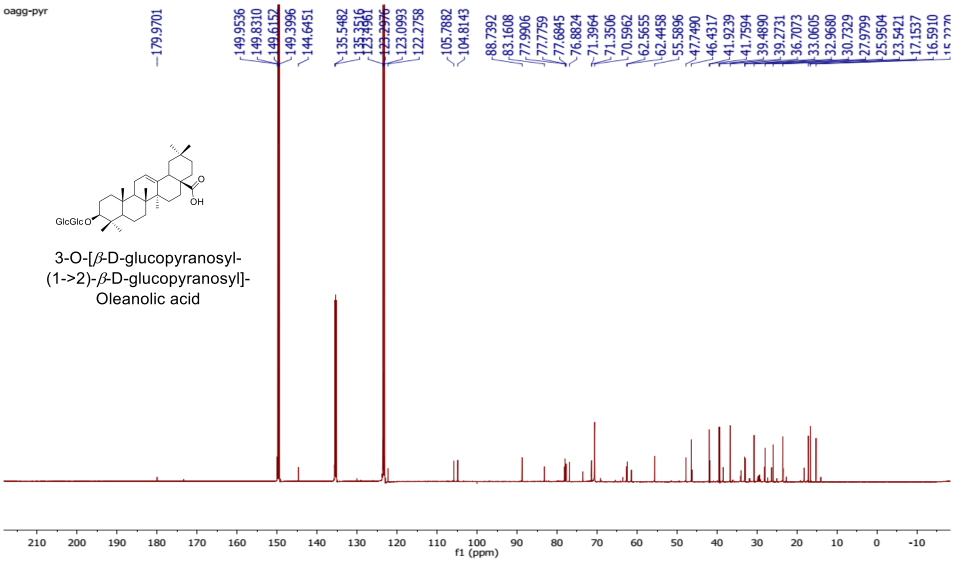
oagg-pyr
GlcGlcO
OH
3-O-[β-D-glucopyranosyl-(1->2)-β-D-glucopyranosyl]-Oleanolic acid
f1 (ppm)

L

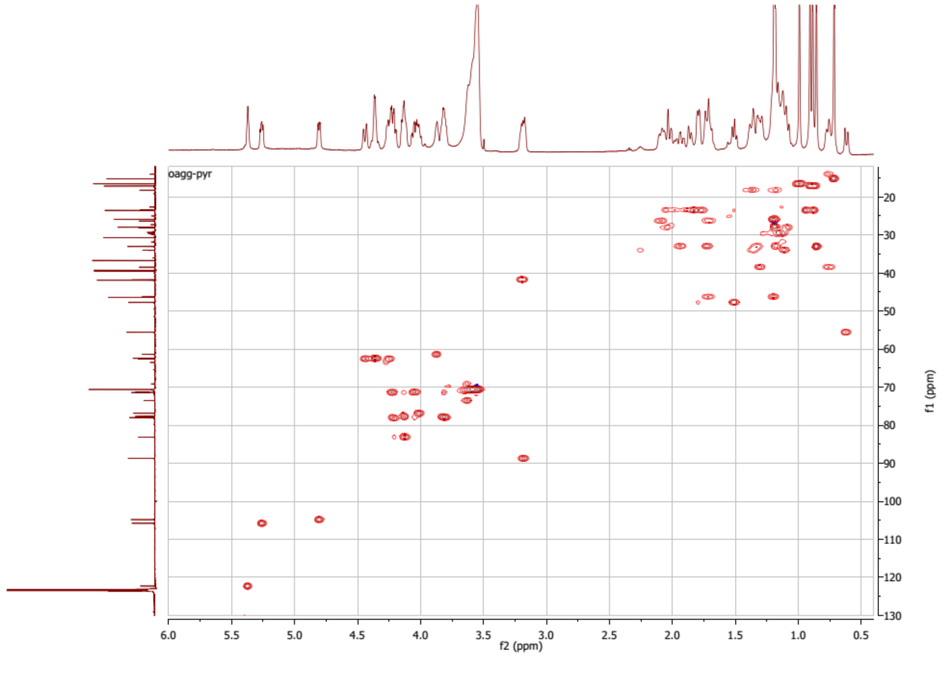
oagg-pyr
f1 (ppm)
f2 (ppm)

M

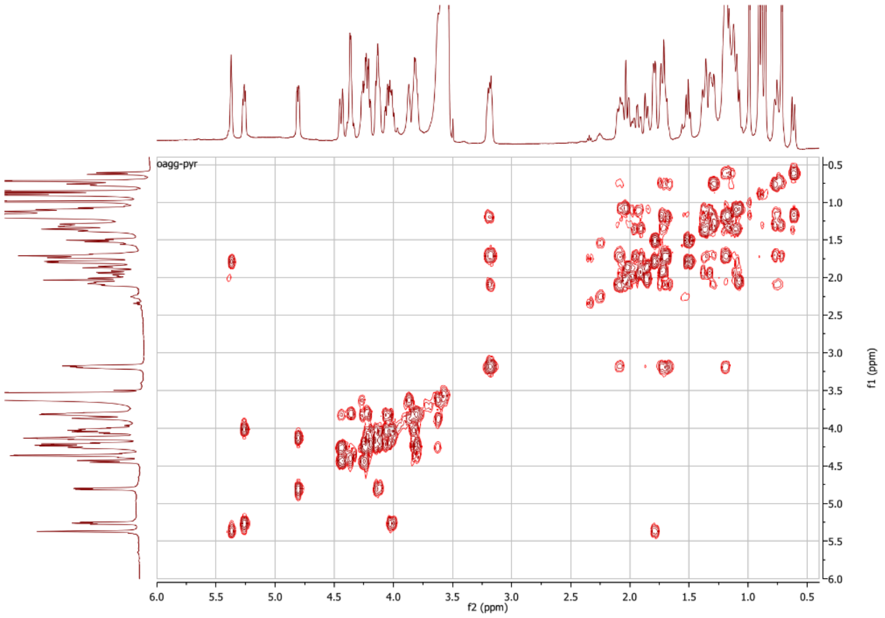

oagg-pyr
f2 (ppm)
f1 (ppm)

N

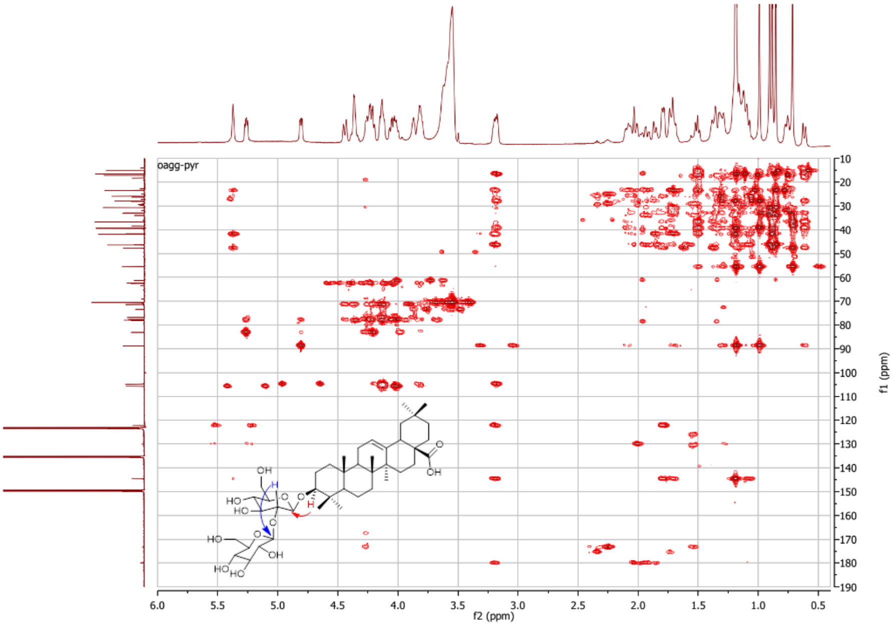

oagg-pyr
f2 (ppm)
f1 (ppm)

O

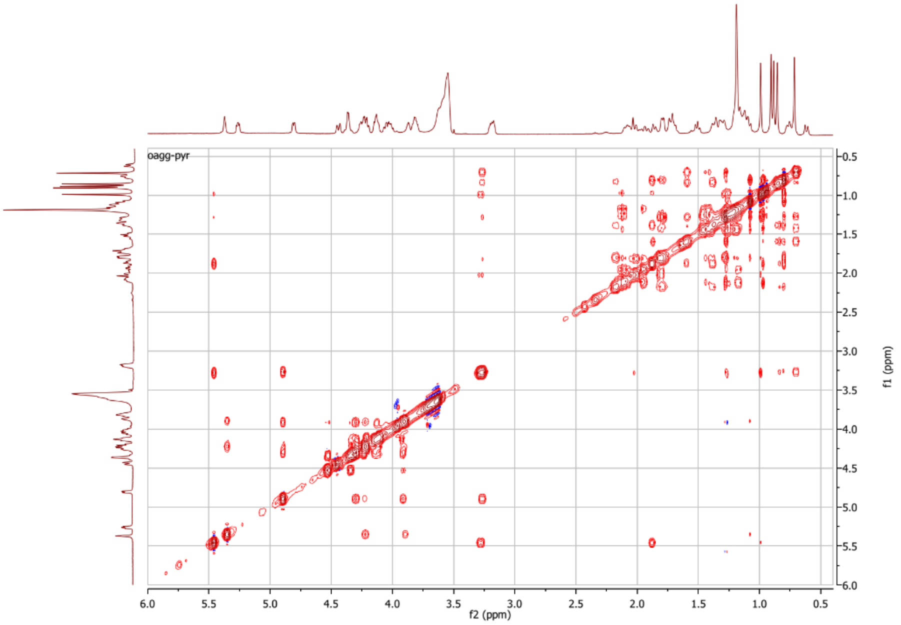

oagg-pyr
f2 (ppm)
f1 (ppm)

Table S1. The statistics of clones and activities of PgUGT94s from the cDNA of a mixed *P. ginseng* sample.

Table S2. The identity matrix of amino acid sequences of PgUGT94s from the cDNA of a mixed *P. ginseng* sample.

Table S3. The statistics of clones and activities of PgUGT94s from the cDNA of a single *P. ginseng* plant.

Table S4. The identity matrix of amino acid sequences of PgUGT94s from the cDNA of a single *P. ginseng* plant.

Table S5. The statistics of clones and activities of PnUGT94s from the cDNA of a single *P. notoginseng* plant.

Table S6. The identity matrix of amino acid sequences of PnUGT94s from the cDNA of a single *P. notoginseng* plant.

Table S7. The statistics of clones and activities of PnUGT94s from the cDNA of *P. notoginseng* leaf of other place.

Table S8. The identity matrix of amino acid sequences of PnUGT94s from the cDNA of *P. notoginseng* leaf of other place.

Table S9. The identity matrix of amino acid sequences of all cloned UGT from *P. ginseng* and *P. notoginseng.*

Table S10. Dataset for construct of *Panax* cDNA database.

Table S11. Data for enzyme activity comparison of UGT94s
